# EDX-SEM-XRF data from selected Precambrian Basement Complex rock samples in part of Southwestern Nigeria

**DOI:** 10.1016/j.dib.2018.09.014

**Published:** 2018-09-08

**Authors:** J.S. Kayode, Y. Yusup, M.N.M. Nawawi, K.S. Ariffin, A.E. Kalil, M.G. Tagwa

**Affiliations:** a Environmental Technology, School of Industrial Technology, Universiti Sains Malaysia, 11800 Pulau-Pinang, Malaysia; b Geophysics Unit, School of Physics, Universiti Sains Malaysia, 11800 Pulau-Pinang, Malaysia; c School of Materials and Mineral Resources Engineering, Universiti Sains Malaysia, 11800 Pulau-Pinang, Malaysia

**Keywords:** EDXA, SEM, XRF, Backscattered electron, Metamorphic rocks, Precambrian Basement Complex

## Abstract

Energy Dispersive X-ray Analysis, EDX mapping, Scanning Electron Microscope, SEM, together with X-ray Fluorescence Analysis, XRF, was carried out to extract the needed data from some metamorphic rock samples in part of the Nigerian Southwestern Precambrian Basement Complex, NSPBC. The foremost aim is to obtain the detail subsurface geological structures of the rocks within the area and to enhanced understanding of the processes and the types of metamorphic evolution in the area. The techniques involved qualitative and quantitative data analysis of the major, minor and radioactive elements present in the samples of rocks analyzed. The data helped to experimentally evaluate the rocks microstructures, and to also explore the development of magmatic and metamorphic mechanisms for the recognition of textual associations in the area. Applications of the EDX, SEM, and XRF data analysis are effortlessly done to determine the varied mixtures of Si, Al, Ca, Fe, K, Mg, and Na, in the presence of O existing in the rocks samples.The data helped in the classification and perceptive of these rocks and it was considered as a necessary tool in the knowledge of the metamorphism and origin of the Basement Complex rocks through measurement of the intensity of the emitted X-ray and its characteristics.

**Specifications table**

**Table:** 

Subject area	*Earth and Planetary Sciences*
More specific subject area	*Mineral Science*
Type of data	*Table, x-ray image, microscopy, text file, graph, and figures*
How data was acquired	EDX mapping and SEM (using Philips CM300 FEG SEM, Great Britain, UK), The XRF (i) Ring Milling Machine, ii) Endecott׳s sieve shaker, iii) RIX 3000, Rigaku, Tokyo, Japan.
Data format	*Raw data.*
Experimental factors	EDX and SEM samples were thoroughly clean with the air pressure cleaner to prevent impurities and contaminations from other samples. The surfaces were polished before mounting on the electron microscope stands. Prior to the XRF analysis, a Standard Ring Mills was used for pulverizing the rock samples into the desired particles size for the machine. This was followed by high-quality Laboratory Mesh Sieve to extract the highest resolution and exceptionally accurate size of the rock samples selected for the XRF data analysis. A 63-micron sieve was selected and used to accurately sieve the ground samples of 100 μm from the ring mill using electrically powered Endecott׳s sieve shaker.
Experimental features	Both the EDX and the SEM machines were interconnected to simultaneously analyze the samples. The samples selected for the XRF were sieved and weighed to obtain the required quantity, (i.e., minimum of 75 μm and 30 g). The samples were subsequently bagged using a cellophane size bag. The stainless-steel sieve and the lid are thoroughly clean with the air pressure cleaner to prevent contaminations from other samples and other materials after each operation. The white plane paper used for the collections of sieved samples was changed after each operation and the place of transferring the samples to the cellophane bag was thoroughly clean with the air pressure cleaner to prevent contaminations of the samples.
Data source location	*Omu-Aran schist belt, Southwestern, Nigeria, spreading across Latitudes N 7° 59′ 45.6′′ and 8° 30′ 22.21′′ and Longitudes E 5° 0′ 1.55′′ and 5° 29′ 57.95′′, bounded by the land area of 3381.12 km^2^*.
Data accessibility	*The data is with this article.*
Related research article	Dai S, Wang X, Zhou Y, Hower Jc, Li D, Chen W, Zhu X, Zou J. Chemical and mineralogical compositions of silicic, mafic, and alkali tonsteins in the late Permian coals from the Songzao Coalfield, Chongqing, Southwest China. J. *Chem. Geo.* (2011). 282**,** 29–44.
	Dai S, Li T, Jiang Y, Ward Cr, Hower Jc, Sun J, Liu J, Song H, Wei J, Li Q. Mineralogical and geochemical compositions of the Pennsylvanian coal in the Hailiushu Mine, Daqingshan Coalfield, Inner Mongolia, China: Implications of sediment-source region and acid hydrothermal solutions. *International J. Coal Geo.* (2015). 137**,** 92–110.
	Dai S, Zhang W, Ward Cr, Seredin Vv, Hower Jc, Li X, Song W, Wang X, Kang H, Zheng L. Mineralogical and geochemical anomalies of late Permian coals from the Fusui Coalfield, Guangxi Province, southern China: influences of terrigenous materials and hydrothermal fluids. *International J. Coal Geo.* (2013). 105**,** 60–84.

**Value of the data**

• The data is useful in the classifications of the chemical composition of each rock sample, which helped in the knowledge of their formations and most importantly the origin and petrogenesis.

• The data helped in the recognition of these rocks; their tectonic settings, understanding of the metamorphic processes and the nature of their hydrothermal fluids as it affects the parent rocks.
• Data of the major, minor and radioactive elements helped in the classification and perceptive of these rocks as necessary tools in the knowledge of the metamorphism and origin of the Nigerian Southwestern Basement Complex rocks.
• Reliable chemical information is now being obtained from SEM, EDX and XRF system to accurately characterized quantitatively the chemical composition of rock samples. The high standard quantitative chemical analysis is rapidly achieved through the X-ray collection in the EDX detector.

## 1 Data

To clarify recognized microstructures of the metamorphic rocks from the study area that comprises of rocks samples characterized by varied mixtures of Si, Al, Ca, Fe, K, Mg, and Na, in the presence of O are effortlessly done with the applications of EDX, SEM, and XRF analysis. Through extensive accessibility, low cost, and versatile transmitted and reflected light microscopy has constantly served as the core of petrographic data collection [1], [2].

**Fig. 1:**
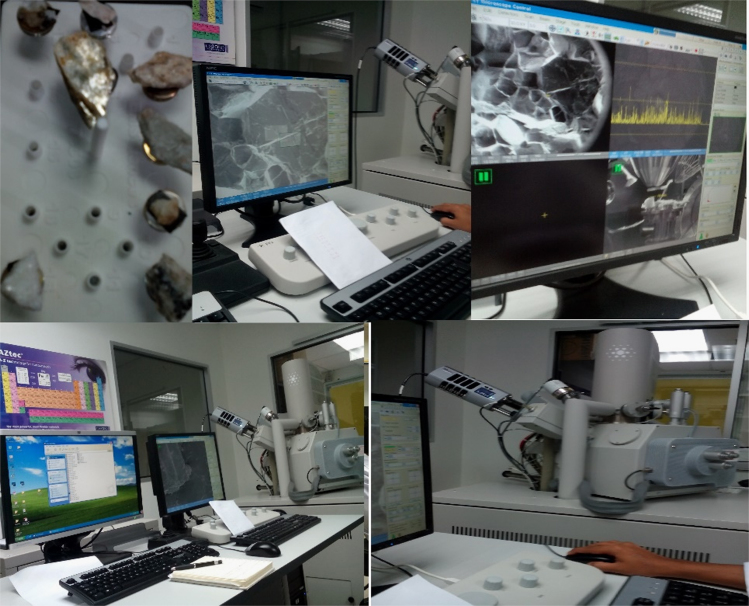
Scanning Electron Microscopy with energy dispersive X-Ray analysis.

EDX is an analytical technique used for the analysis of all the elements present in each of the samples. The interaction between the samples and the X-ray excitation was used in the accurate characterization of the samples. Both the EDX and the SEM machines were interconnected to simultaneously analyze the samples as shown in Fig. 1. The EDX and SEM analysis of few selected rocks samples from the study area was carried out at the Universiti Sains Malaysia Centre for Global Archaeological Research Earth Material Characterisation Laboratory. However, all the preparations and the XRF analysis were carried out at the School of Materials and Mineral Resources Engineering, Engineering Campus, Universiti Sains Malaysia [1], [3], [4].

## 2 Experimental design, materials and methods

**Fig. 2:**
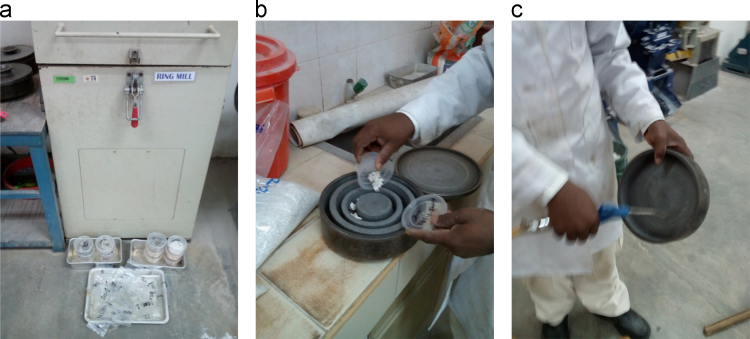
Operation and processing of rock samples on the Standard Ring Mills.

Preparation of the rock samples collected from the study area for XRF analysis involved crushing of the rocks to smaller sizes for the Ring Milling Machine to grind the materials for subsequent operations by other machines. Fig. 2 shows the process of crushing the rocks with the Ring Milling Machine with adequate precautions to ensure non-contaminations of the samples with other samples or impurities.

**Fig. 3:**
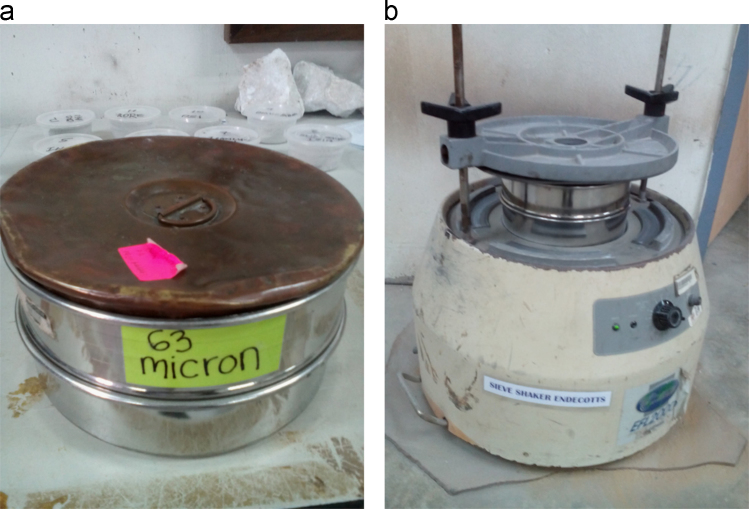
(a) Laboratory mesh 63-micron stainless steel Sieve, (b) Endecott׳s Power Sieve Shaker.

A high-quality Laboratory Mesh Sieve was used to extract the highest resolution and exceptionally accurate size of the particles of rock samples for the XRF analysis. A 63-micron sieve was selected and used to accurately sieve the ground samples of 100 μm from the Ring Mill using electrically powered Endecott׳s sieve shaker. To maintain the highest precision as much as possible and prevents contaminations and impurities to the samples, the sieve, the stainless-steel pan and the lid were thoroughly clean after each use with the air pressure cleaner. The separate plain white paper was used for each of the samples in the process of transferring from the stainless-steel pan to the cellophane bag. Operations of the 63-micron stainless steel sieve and the Endecott׳s power sieve shaker is as presented in Fig. 3.

**Fig. 4:**
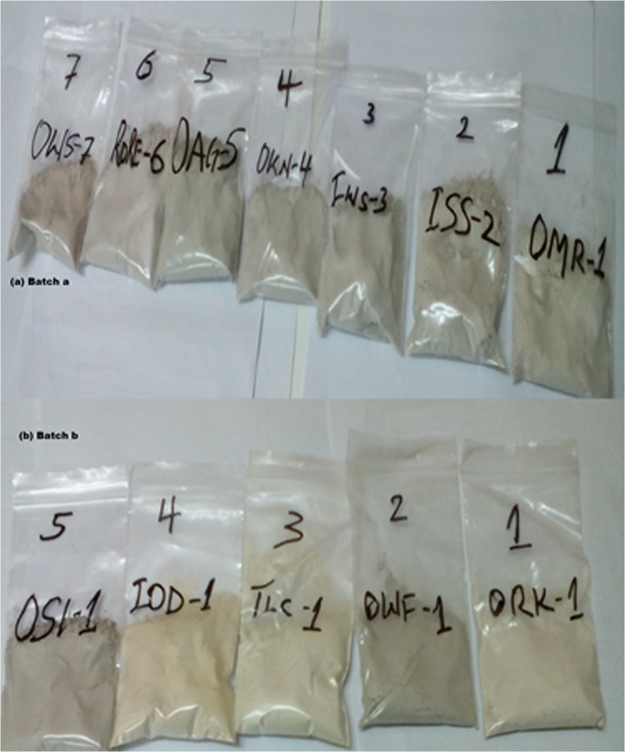
Prepared 30 g samples of 75 μm for the XRF analysis.

Fig. 4 shows the 63-micron generated samples for the XRF analysis. The samples were sieved and weighed to obtain the required quantity of 30 g minimum of 75 μm needed for the data.

**Fig. 5:**
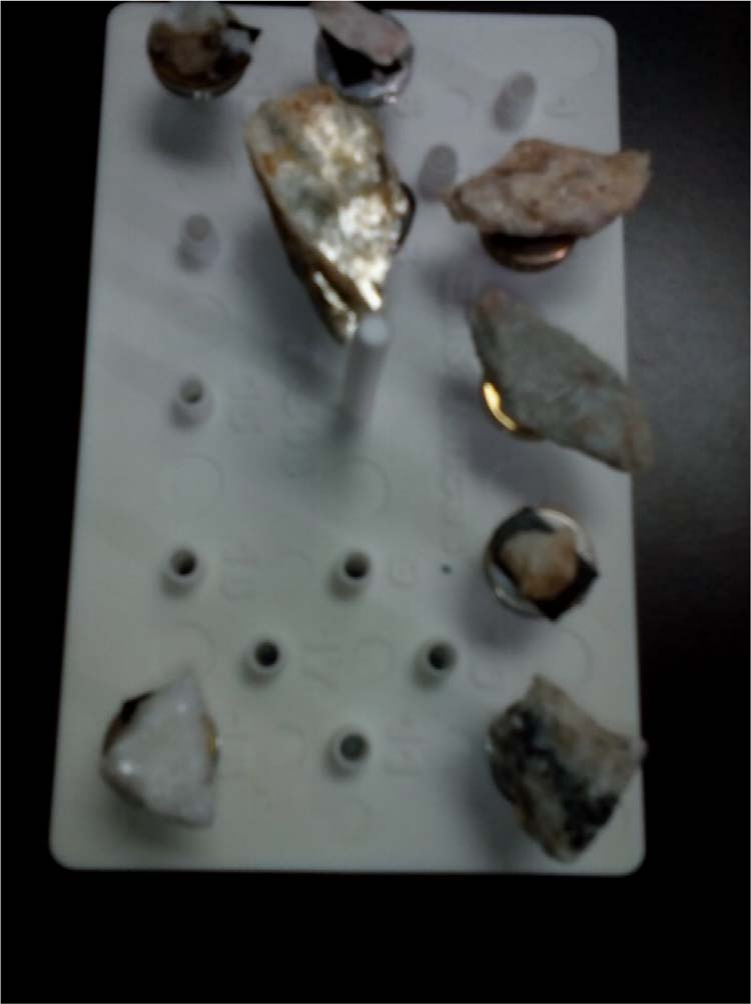
Prepared rocks samples mounted on the machine stands for EDX and SEM analysis.

Preparation of the rocks samples collected for the EDX and SEM analysis involved cutting of the required quantity, polishing and cleaned with air pressure cleaner before mounting on the machine stands as shown in Fig. 5.
