# Supplementary material for: EDX-SEM-XRF data from selected Precambrian Basement Complex rock samples in part of Southwestern Nigeria
Source: Data Brief. 2018 Sep 8;20:1525–31. doi: 10.1016/j.dib.2018.09.014 (PMC6153388; doi:10.1016/j.dib.2018.09.014)
Supplement: Supplementary file 2 — Supplementary material [file mmc2.doc]

MAKMAL PENCIRIAN BAHAN BUMI (SEM/EDX/WDX)


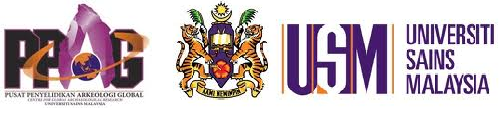


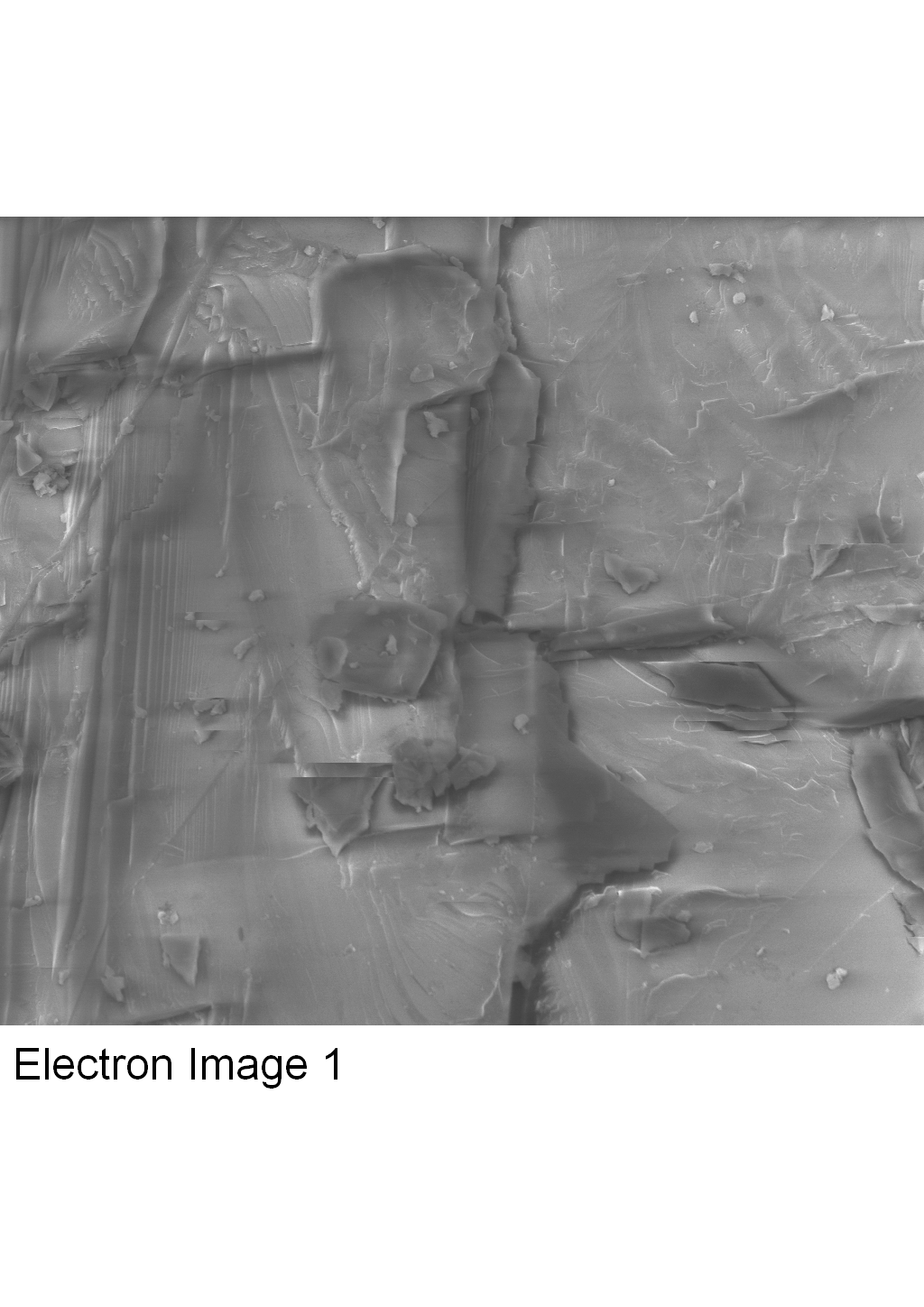

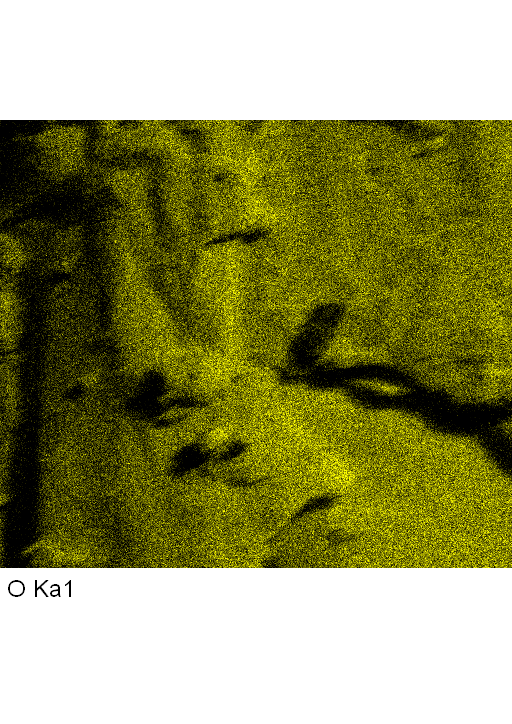

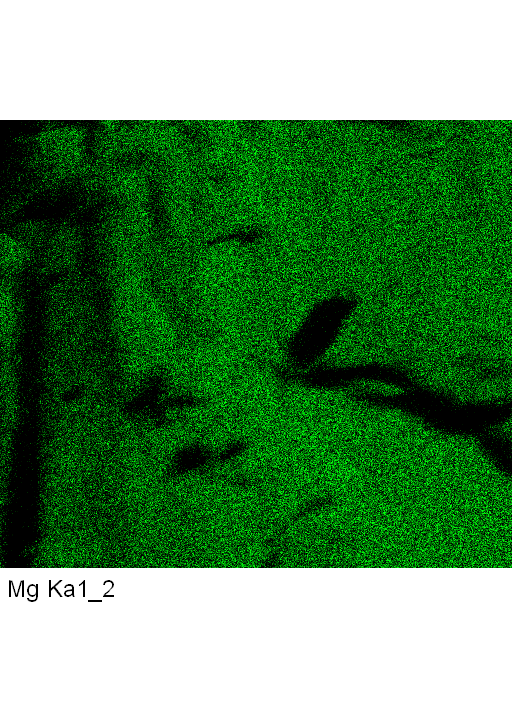

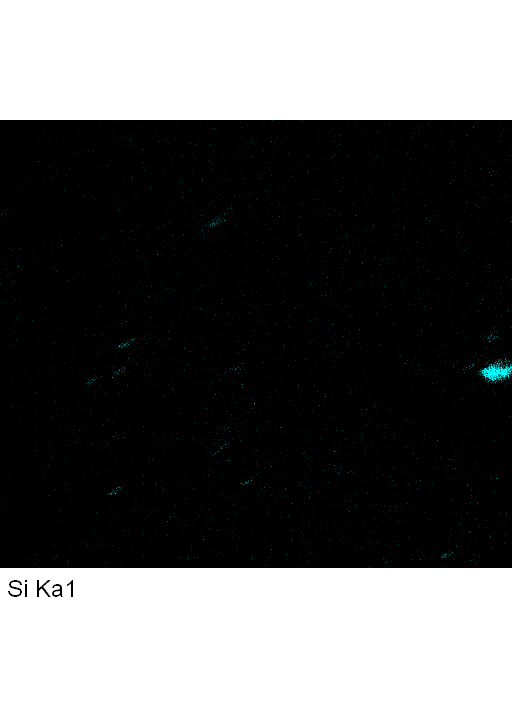

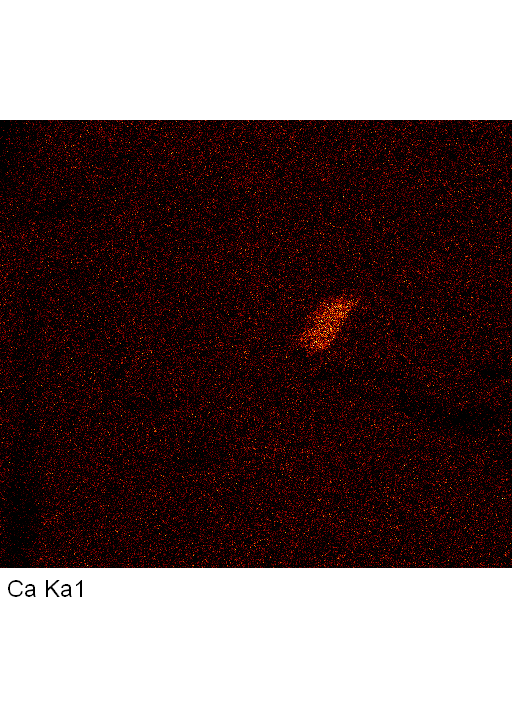

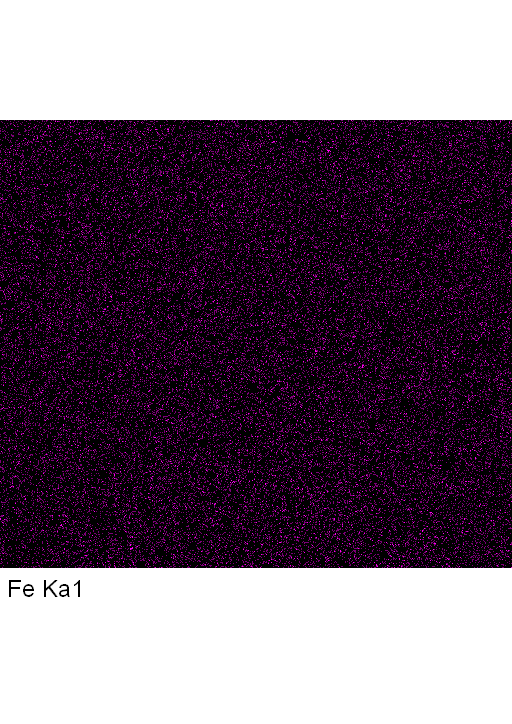


Sample: Sample 1

Type: Default

ID:

Spectrum processing :

Peaks possibly omitted : 2.280, 2.617 keV

Processing option : All elements analyzed (Normalised)

Number of iterations = 5

Standard :

C CaCO3 1-Jun-1999 12:00 AM

O SiO2 1-Jun-1999 12:00 AM

Mg MgO 1-Jun-1999 12:00 AM

Si SiO2 1-Jun-1999 12:00 AM

Ca Wollastonite 1-Jun-1999 12:00 AM

Fe FeS2 2-May-2012 05:20 PM

| Element | Weight% | Atomic% |  |
| --- | --- | --- | --- |
|  |  |  |  |
| C K | 14.63 | 21.46 |  |
| O K | 57.28 | 63.09 |  |
| Mg K | 10.81 | 7.83 |  |
| Si K | 0.15 | 0.09 |  |
| Ca K | 17.03 | 7.49 |  |
| Fe K | 0.10 | 0.03 |  |
|  |  |  |  |
| Totals | 100.00 |  |  |

08/03/2017 16:22:18
