# Supplementary material for: EDX-SEM-XRF data from selected Precambrian Basement Complex rock samples in part of Southwestern Nigeria
Source: Data Brief. 2018 Sep 8;20:1525–31. doi: 10.1016/j.dib.2018.09.014 (PMC6153388; doi:10.1016/j.dib.2018.09.014)
Supplement: Supplementary file 3 — Supplementary material [file mmc3.doc]

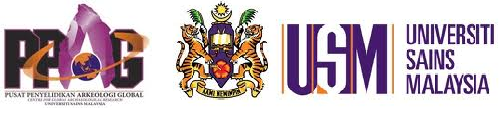

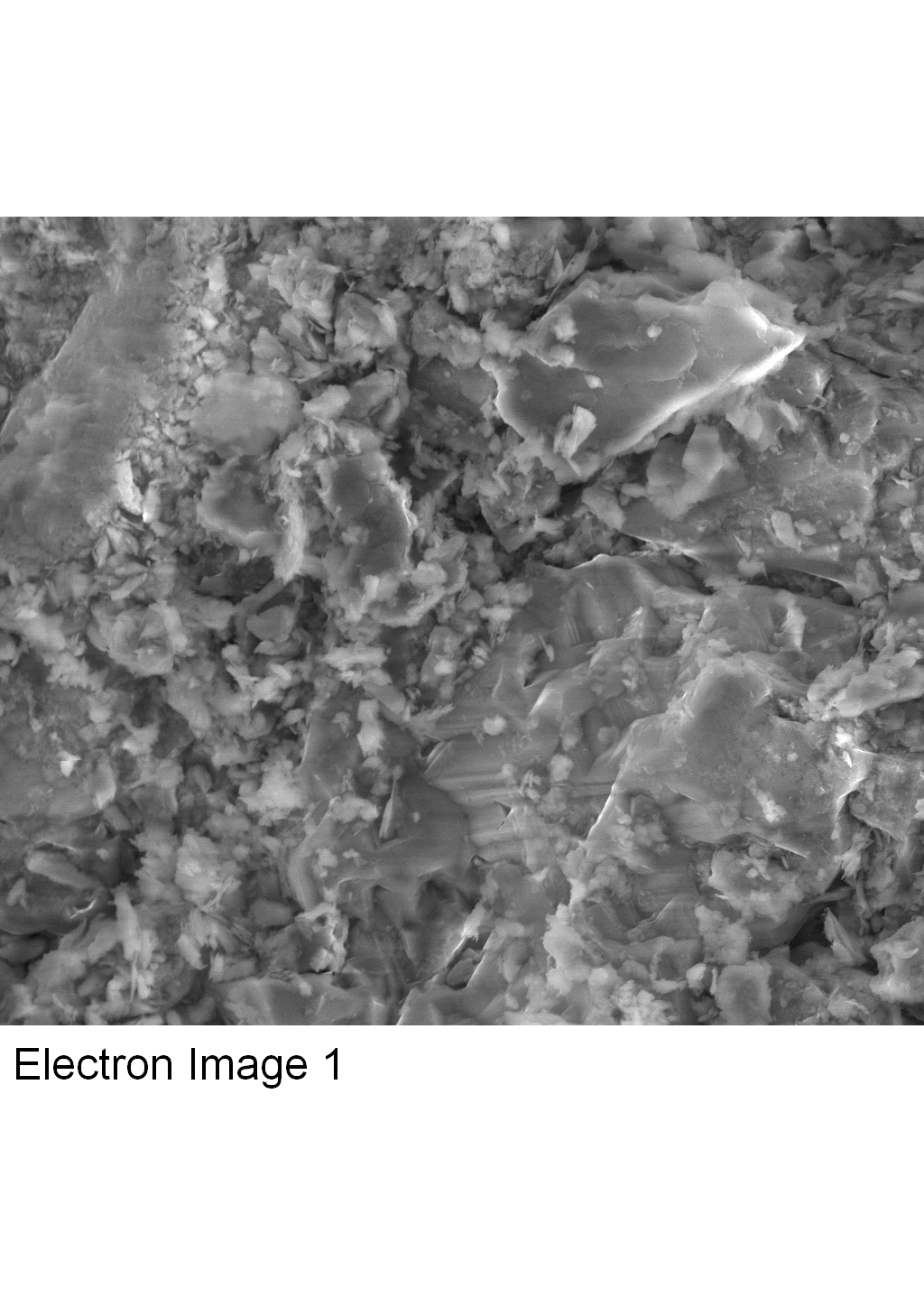

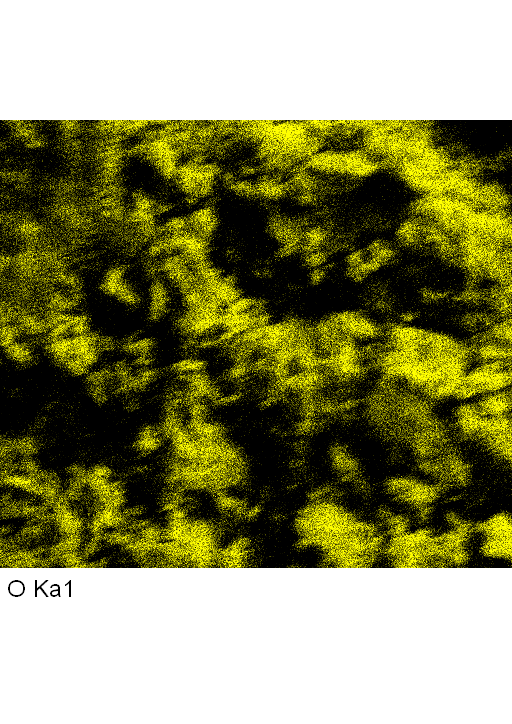

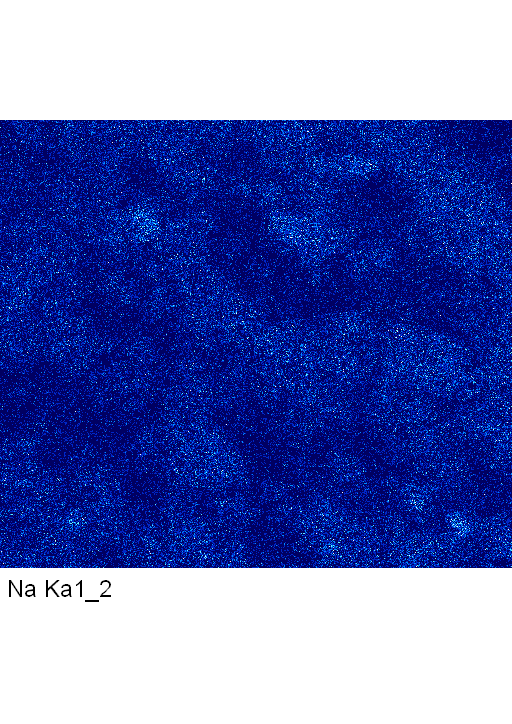

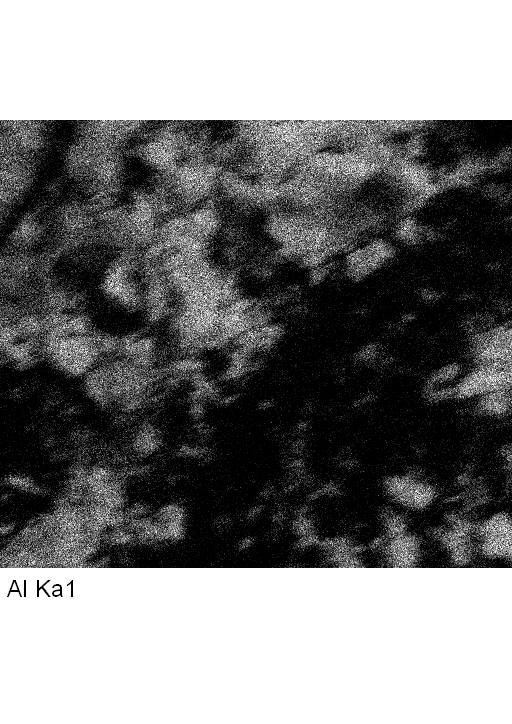

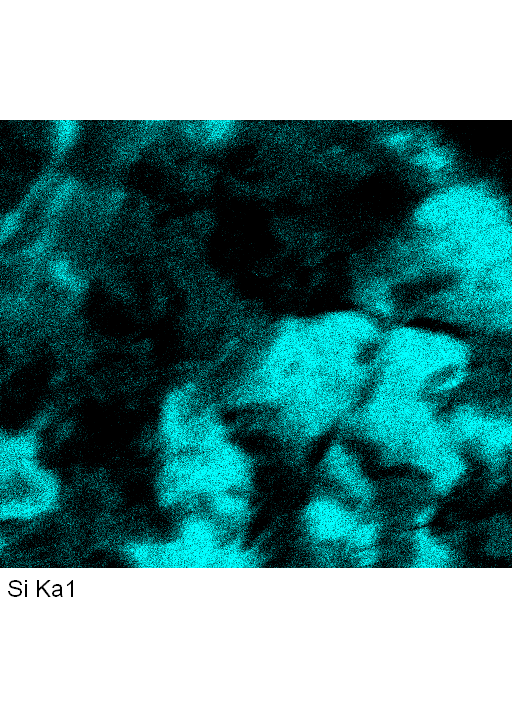

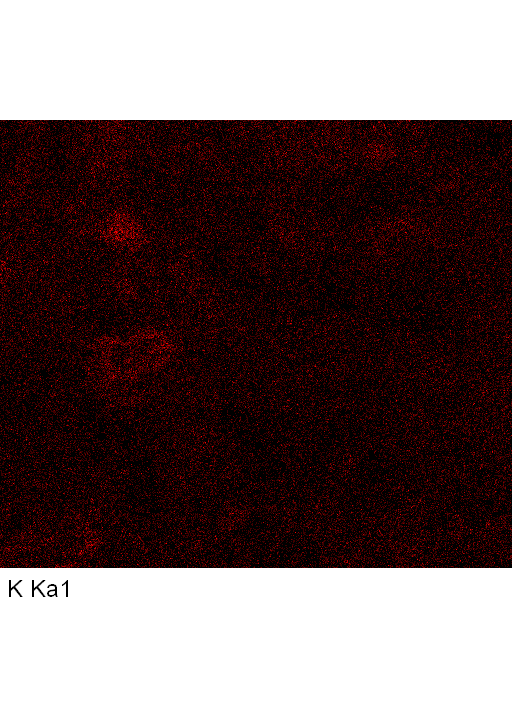

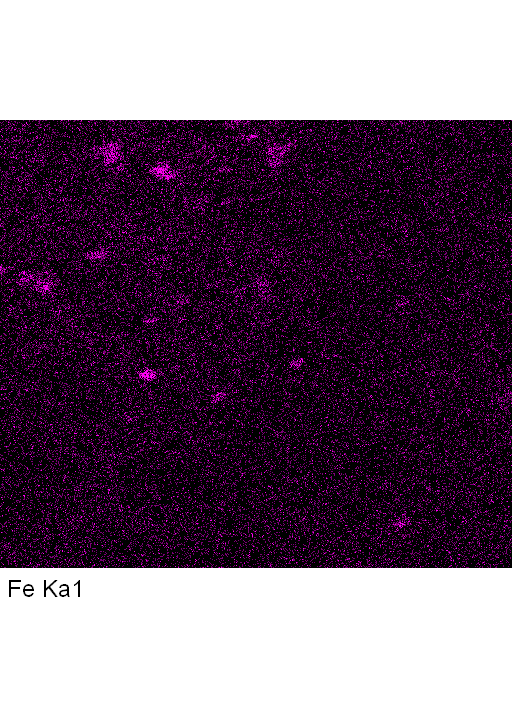


MAKMAL PENCIRIAN BAHAN BUMI (SEM/EDX/WDX)

08/03/2017 18:00:54

Sample: Sample 2

Type: Default

ID:

Spectrum processing :

Peak possibly omitted : 2.626 keV

Processing option : All elements analyzed (Normalised)

Number of iterations = 3

Standard :

C CaCO3 1-Jun-1999 12:00 AM

O SiO2 1-Jun-1999 12:00 AM

Na Albite 1-Jun-1999 12:00 AM

Al Al2O3 1-Jun-1999 12:00 AM

Si SiO2 1-Jun-1999 12:00 AM

K MAD-10 Feldspar 1-Jun-1999 12:00 AM

Fe FeS2 2-May-2012 05:20 PM

| Element | Weight% | Atomic% |  |
| --- | --- | --- | --- |
|  |  |  |  |
| C K | 1.23 | 1.99 |  |
| O K | 56.36 | 68.59 |  |
| Na K | 0.15 | 0.12 |  |
| Al K | 9.91 | 7.15 |  |
| Si K | 31.41 | 21.78 |  |
| K K | 0.27 | 0.14 |  |
| Fe K | 0.67 | 0.23 |  |
|  |  |  |  |
| Totals | 100.00 |  |  |
