# Supplementary material for: EDX-SEM-XRF data from selected Precambrian Basement Complex rock samples in part of Southwestern Nigeria
Source: Data Brief. 2018 Sep 8;20:1525–31. doi: 10.1016/j.dib.2018.09.014 (PMC6153388; doi:10.1016/j.dib.2018.09.014)
Supplement: Supplementary file 4 — Supplementary material [file mmc4.doc]

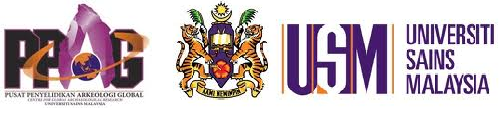

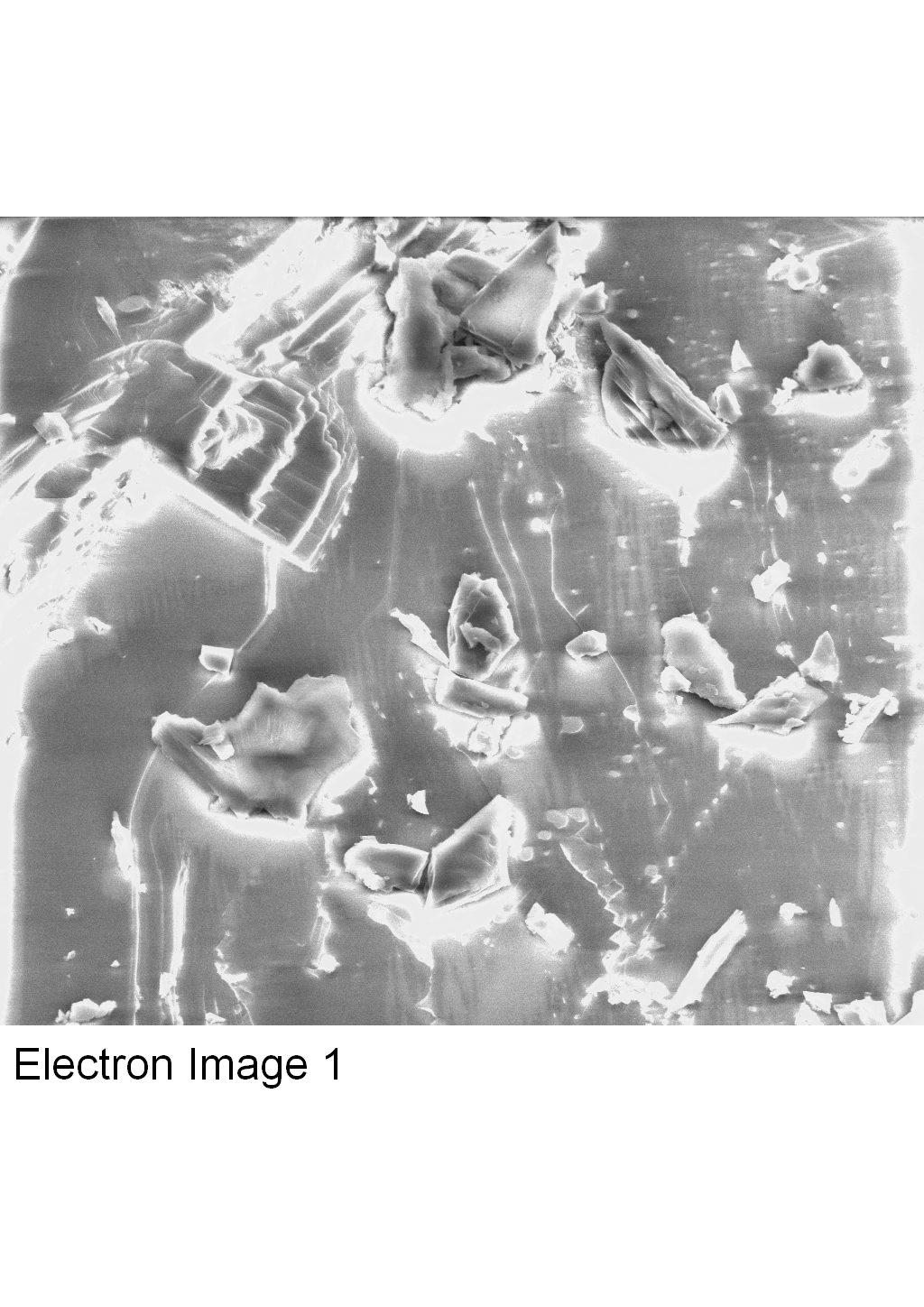

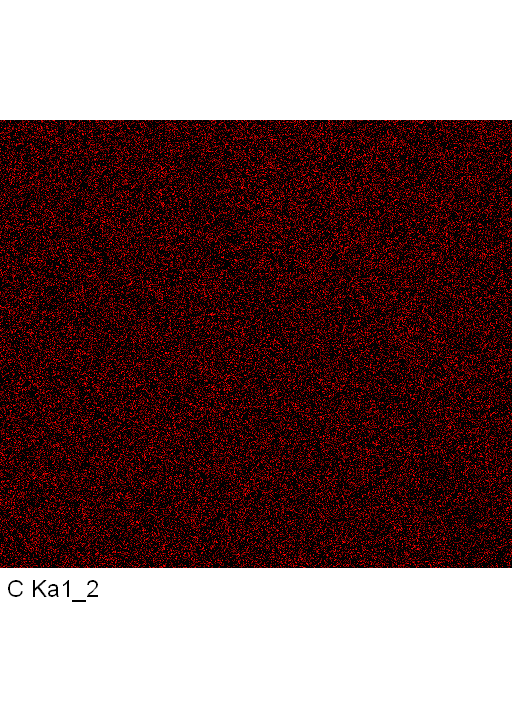

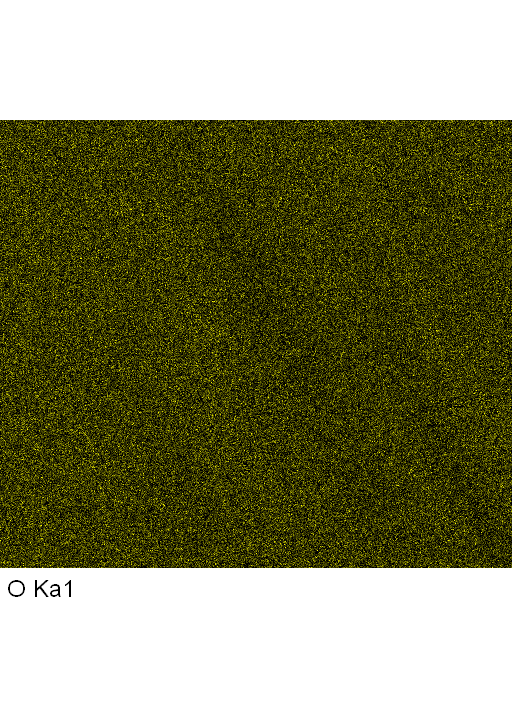

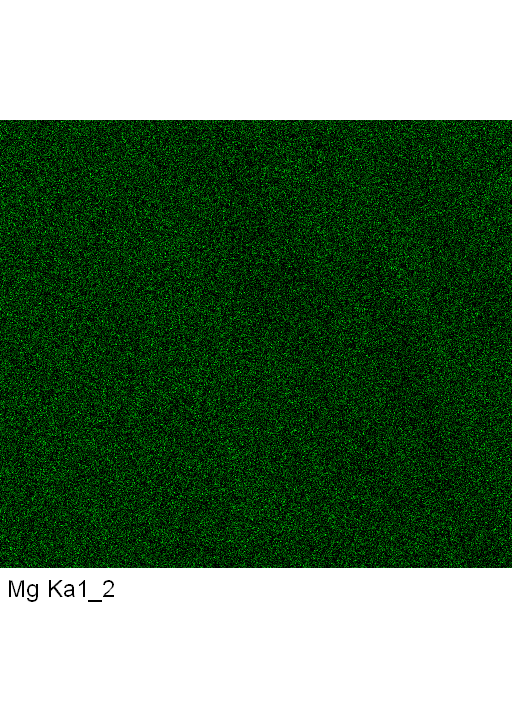

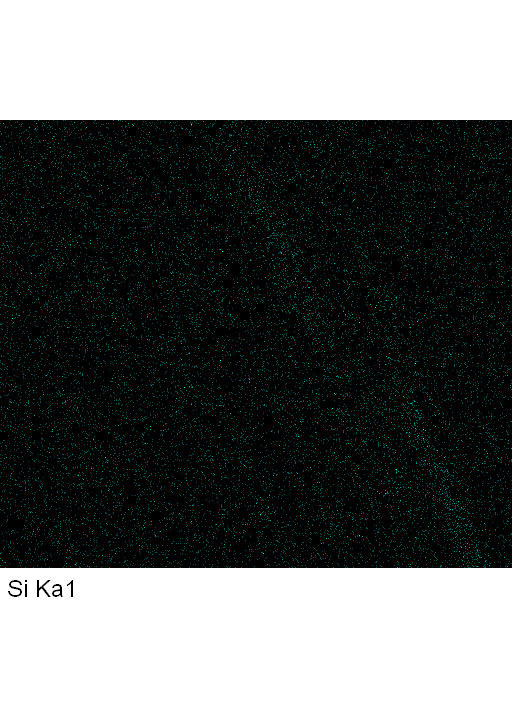

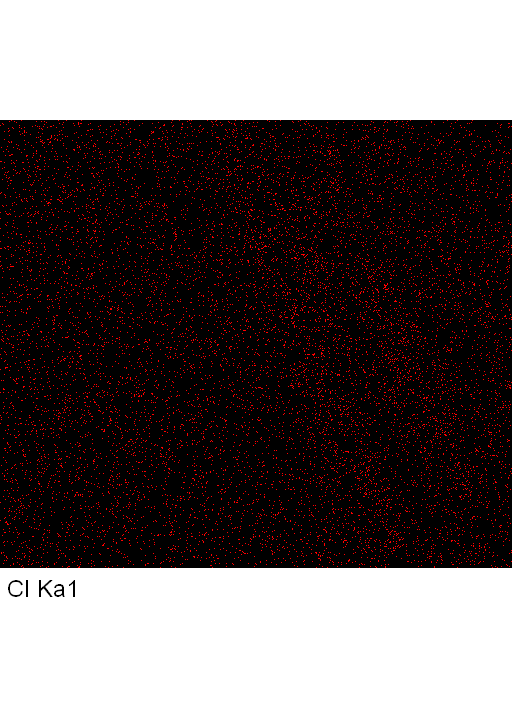

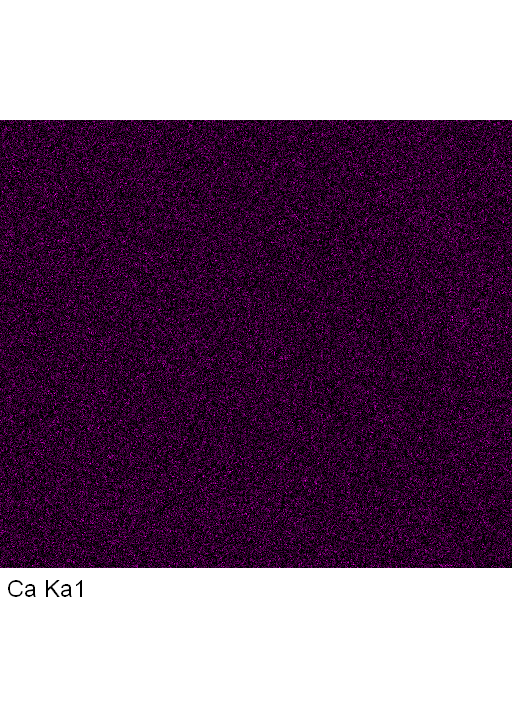

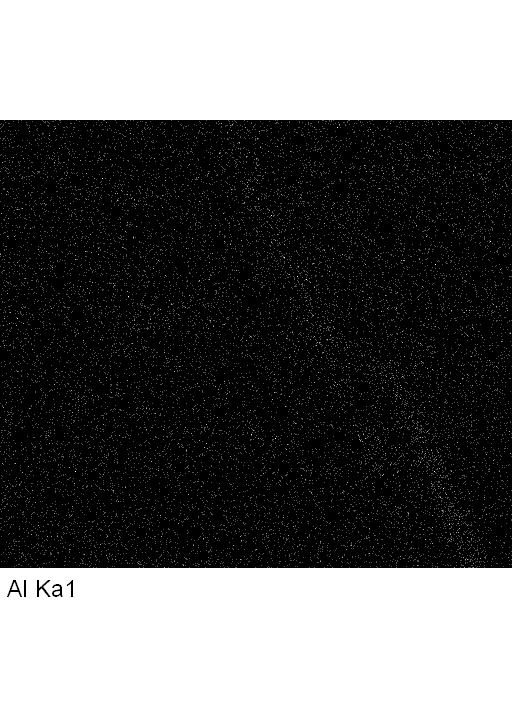


MAKMAL PENCIRIAN BAHAN BUMI (SEM/EDX/WDX)

20/02/2017 12:27:59

Sample: Sample 4

Type: Default

ID:

Spectrum processing :

No peaks omitted

Processing option : Oxygen by stoichiometry (Normalised)

Number of iterations = 2

Standard :

C CaCO3 1-Jun-1999 12:00 AM

Mg MgO 1-Jun-1999 12:00 AM

Si SiO2 1-Jun-1999 12:00 AM

Cl KCl 1-Jun-1999 12:00 AM

Ca Wollastonite 1-Jun-1999 12:00 AM

Fe FeS2 2-May-2012 05:20 PM

| Element | Weight% | Atomic% | Compd% | Formula |  |
| --- | --- | --- | --- | --- | --- |
|  |  |  |  |  |  |
| C K | 15.45 | 22.66 | 56.62 | CO2 |  |
| Mg K | 11.64 | 8.43 | 19.30 | MgO |  |
| Si K | 0.16 | 0.10 | 0.34 | SiO2 |  |
| Cl K | 0.13 | 0.07 | 0.00 |  |  |
| Ca K | 16.75 | 7.36 | 23.44 | CaO |  |
| Fe K | 0.14 | 0.04 | 0.18 | FeO |  |
| O | 55.73 | 61.35 |  |  |  |
| Totals | 100.00 |  |  |  |  |
