# Supplementary material for: EDX-SEM-XRF data from selected Precambrian Basement Complex rock samples in part of Southwestern Nigeria
Source: Data Brief. 2018 Sep 8;20:1525–31. doi: 10.1016/j.dib.2018.09.014 (PMC6153388; doi:10.1016/j.dib.2018.09.014)
Supplement: Supplementary file 5 — Supplementary material [file mmc5.doc]

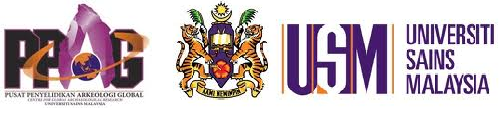

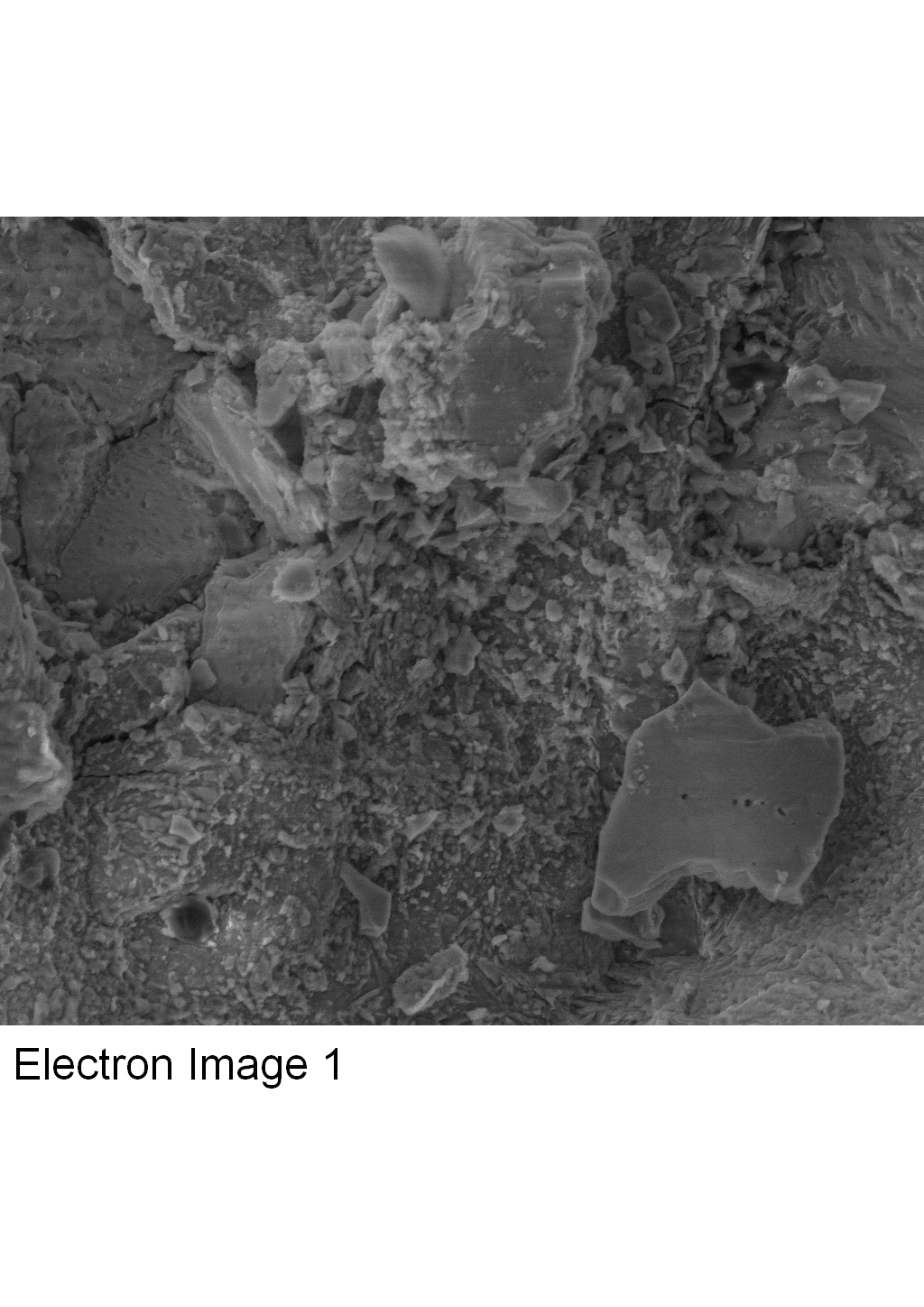

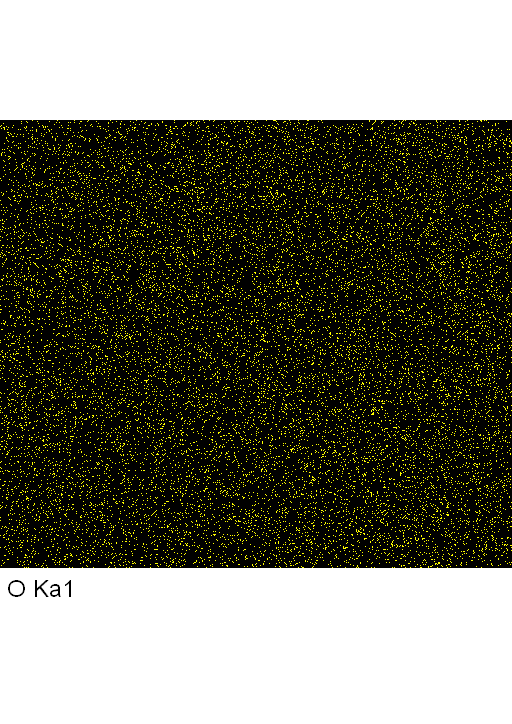

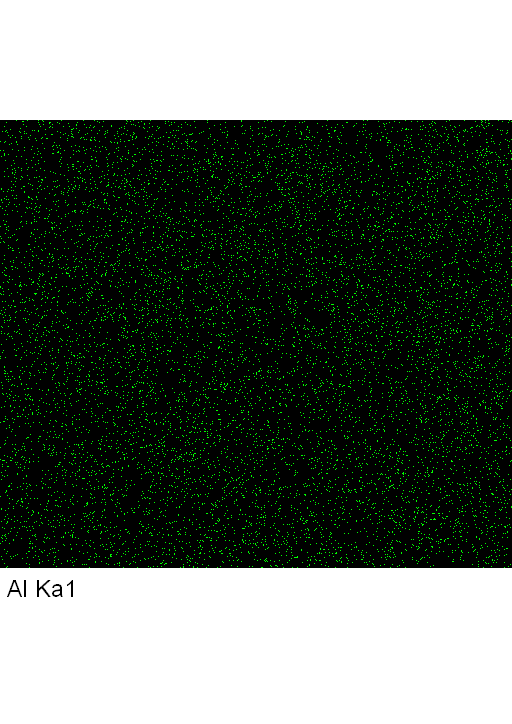

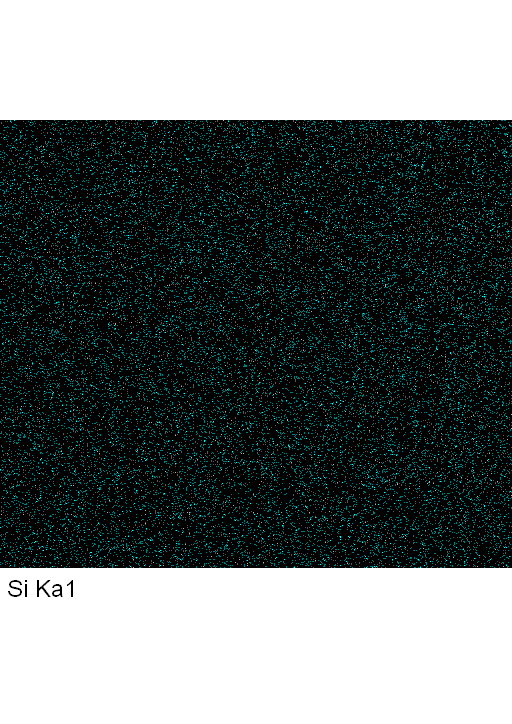

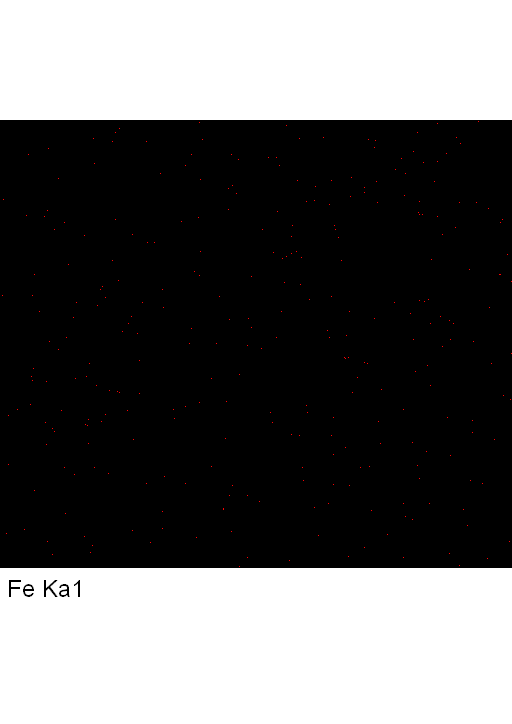


MAKMAL PENCIRIAN BAHAN BUMI (SEM/EDX/WDX)

20/02/2017 15:01:18

Sample: Sample 5

Type: Default

ID:

Spectrum processing :

No peaks omitted

Processing option : Oxygen by stoichiometry (Normalised)

Number of iterations = 1

Standard :

C CaCO3 1-Jun-1999 12:00 AM

Al Al2O3 1-Jun-1999 12:00 AM

Si SiO2 1-Jun-1999 12:00 AM

Fe FeS2 2-May-2012 05:20 PM

| Element | Weight% | Atomic% | Compd% | Formula |  |
| --- | --- | --- | --- | --- | --- |
|  |  |  |  |  |  |
| C K | 13.09 | 18.65 | 47.98 | CO2 |  |
| Al K | 5.64 | 3.58 | 10.65 | Al2O3 |  |
| Si K | 19.06 | 11.61 | 40.77 | SiO2 |  |
| Fe K | 0.47 | 0.14 | 0.60 | FeO |  |
| O | 61.74 | 66.02 |  |  |  |
| Totals | 100.00 |  |  |  |  |
