# Supplementary material for: EDX-SEM-XRF data from selected Precambrian Basement Complex rock samples in part of Southwestern Nigeria
Source: Data Brief. 2018 Sep 8;20:1525–31. doi: 10.1016/j.dib.2018.09.014 (PMC6153388; doi:10.1016/j.dib.2018.09.014)
Supplement: Supplementary file 6 — Supplementary material [file mmc6.doc]

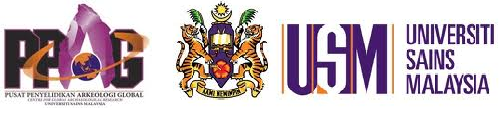

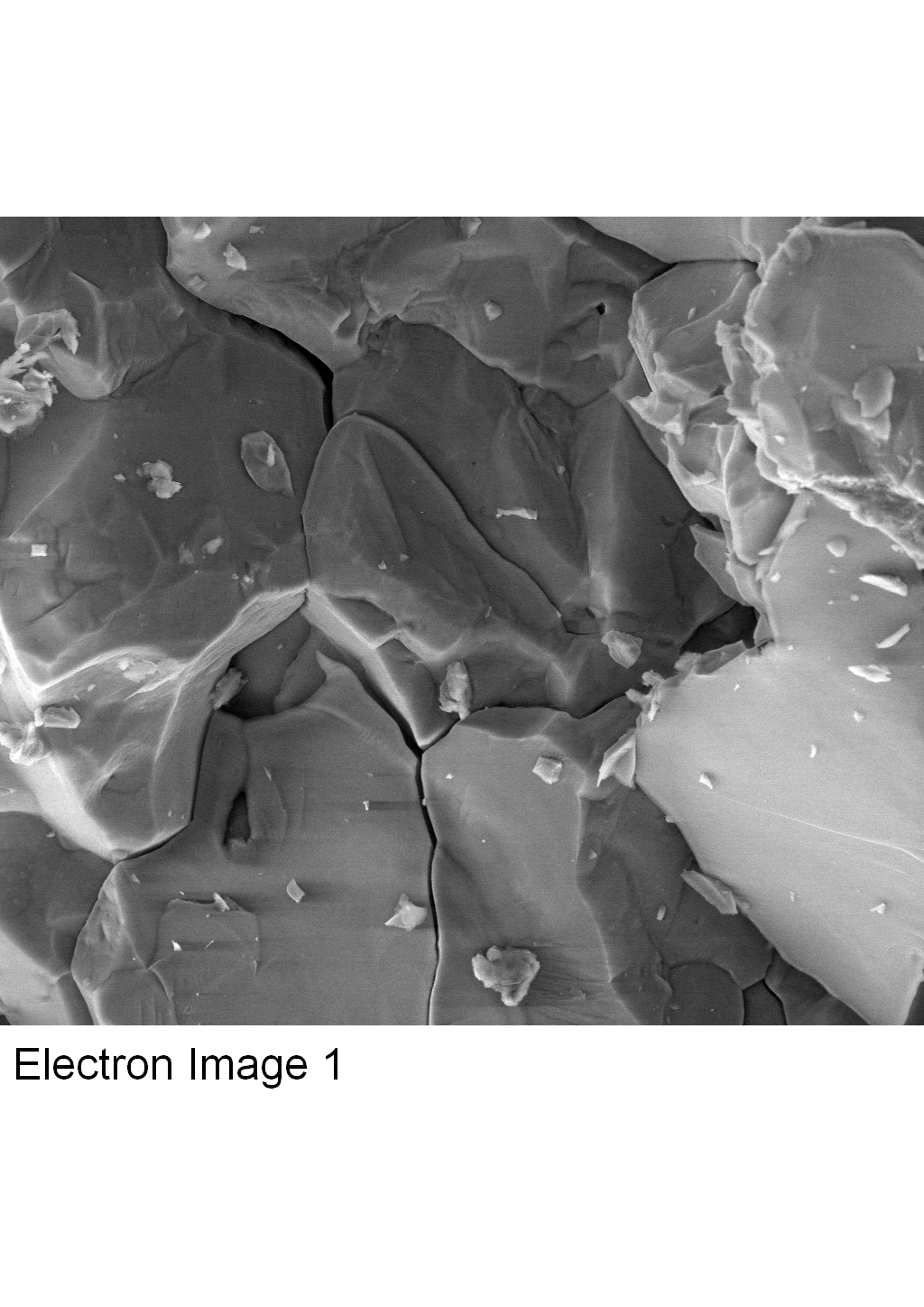

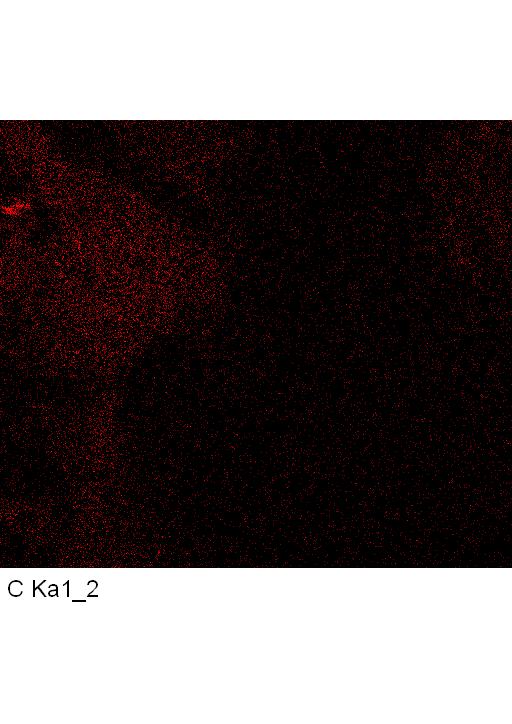

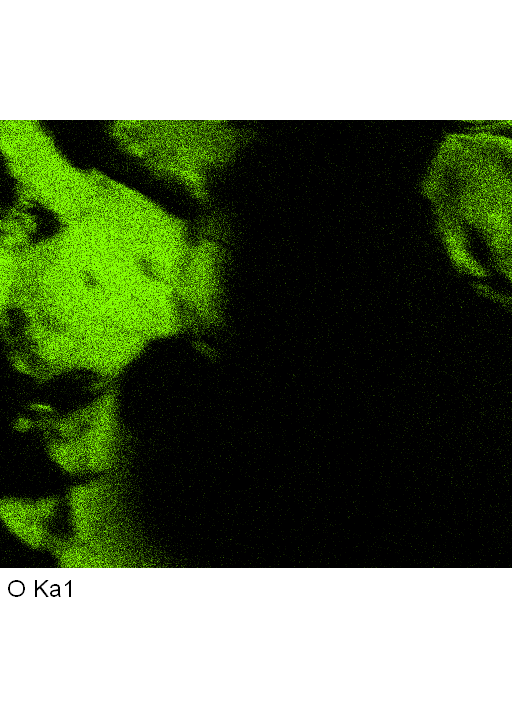

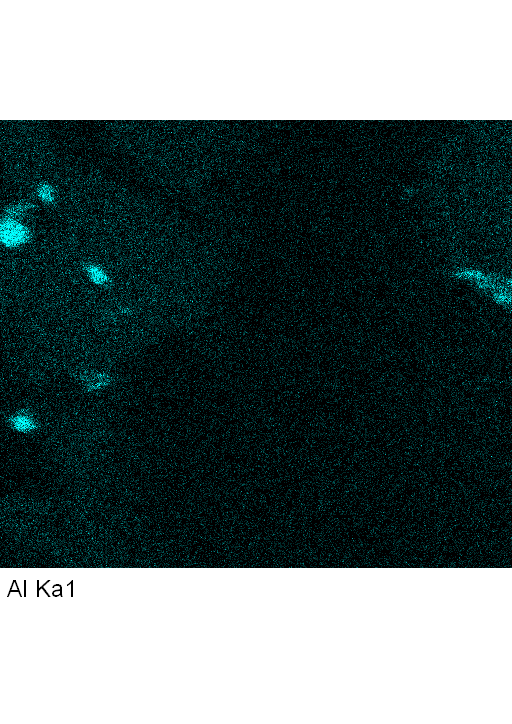

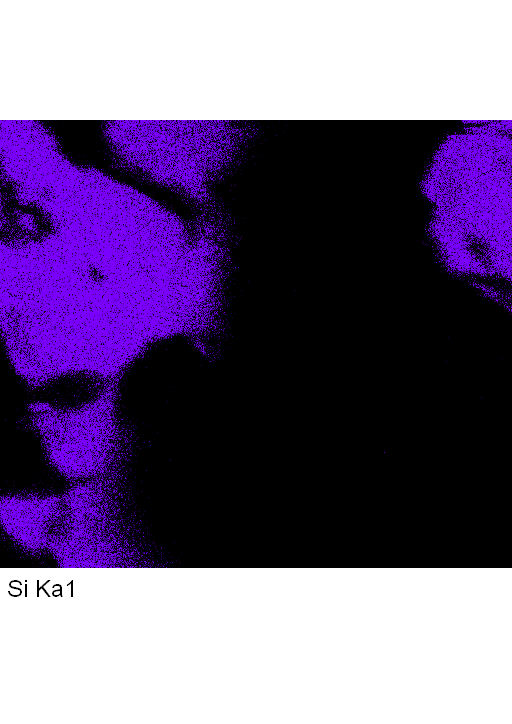


MAKMAL PENCIRIAN BAHAN BUMI (SEM/EDX/WDX)

24/03/2017 11:55:38

Sample: Sample 6

Type: Default

ID:

Spectrum processing :

No peaks omitted

Processing option : All elements analyzed (Normalised)

Number of iterations = 2

Standard :

C CaCO3 1-Jun-1999 12:00 AM

O SiO2 1-Jun-1999 12:00 AM

Al Al2O3 1-Jun-1999 12:00 AM

Si SiO2 1-Jun-1999 12:00 AM

| Element | Weight% | Atomic% |  |
| --- | --- | --- | --- |
|  |  |  |  |
| C K | 2.25 | 3.65 |  |
| O K | 54.36 | 66.21 |  |
| Al K | 1.64 | 1.18 |  |
| Si K | 41.75 | 28.96 |  |
|  |  |  |  |
| Totals | 100.00 |  |  |
