# Supplementary material for: EDX-SEM-XRF data from selected Precambrian Basement Complex rock samples in part of Southwestern Nigeria
Source: Data Brief. 2018 Sep 8;20:1525–31. doi: 10.1016/j.dib.2018.09.014 (PMC6153388; doi:10.1016/j.dib.2018.09.014)
Supplement: Supplementary file 7 — Supplementary material [file mmc7.doc]

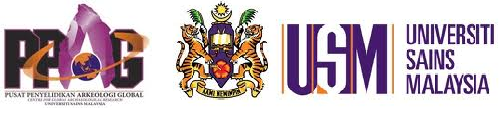

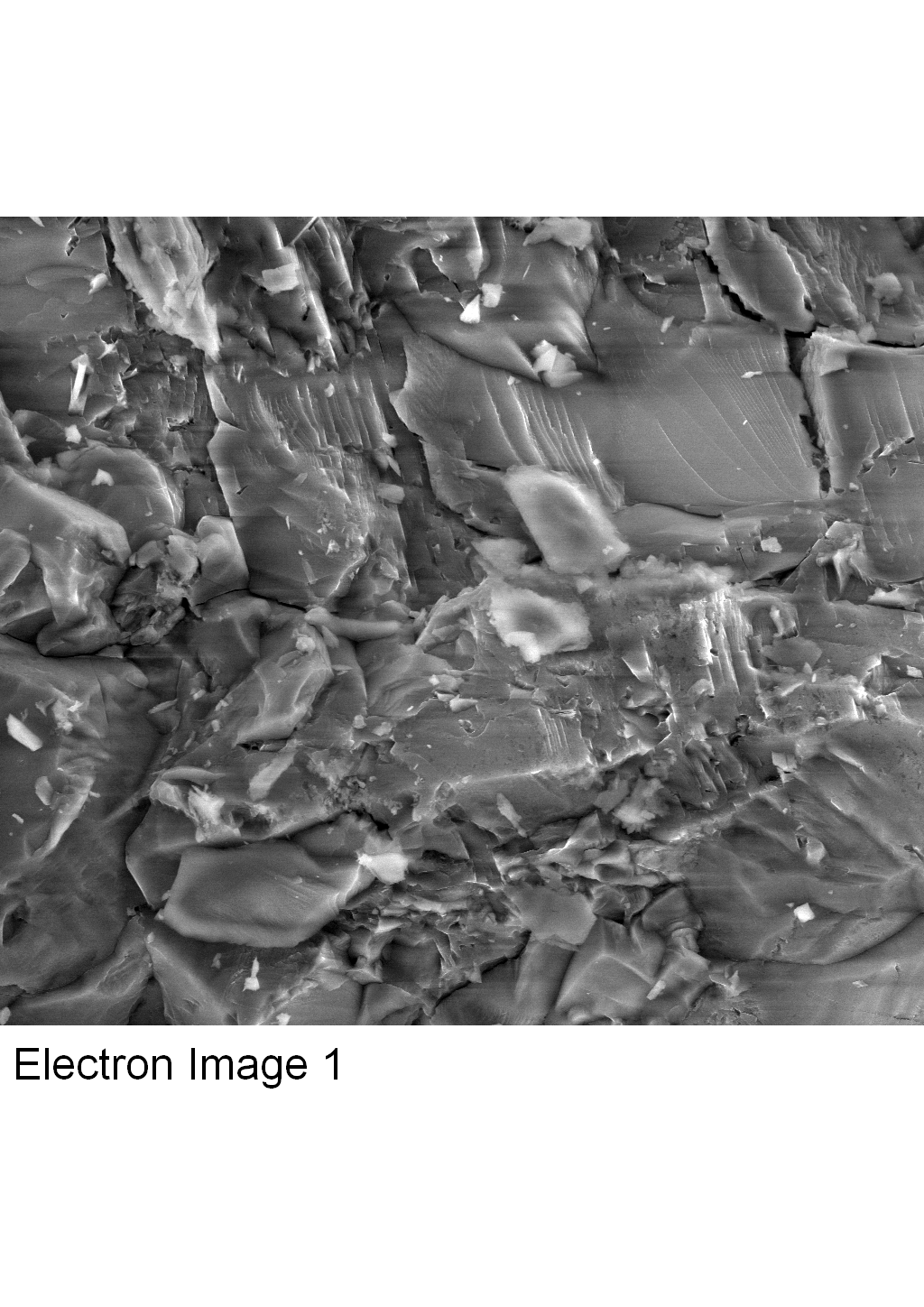

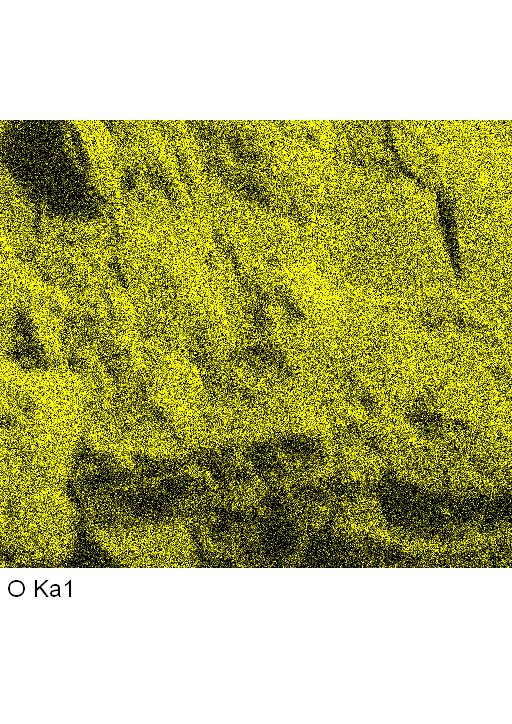

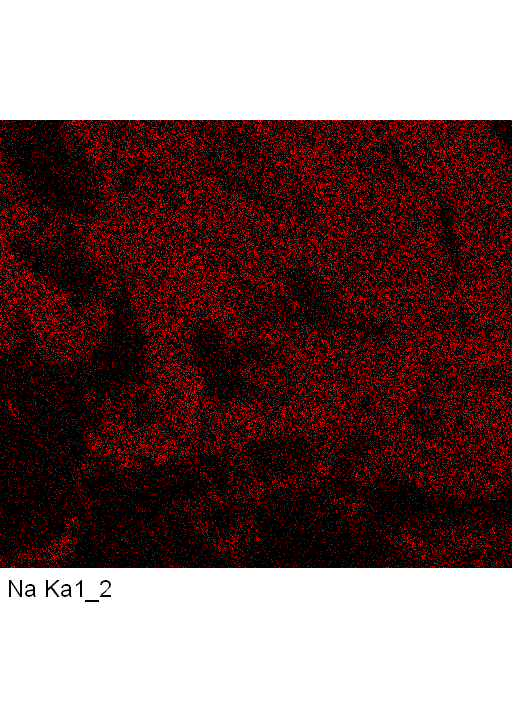

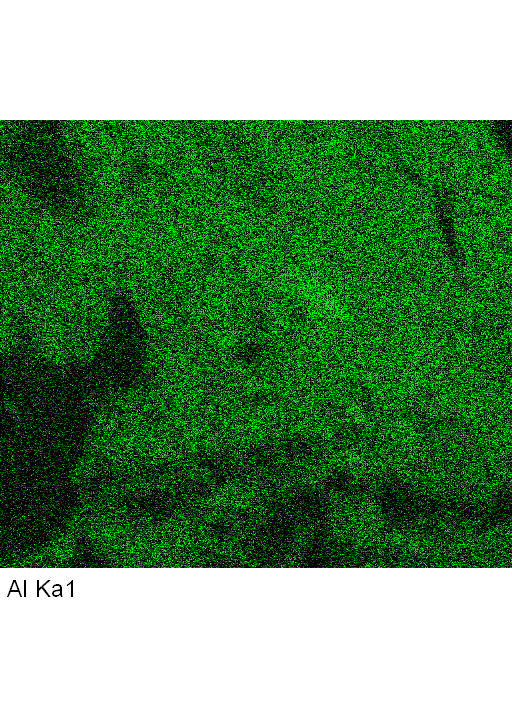

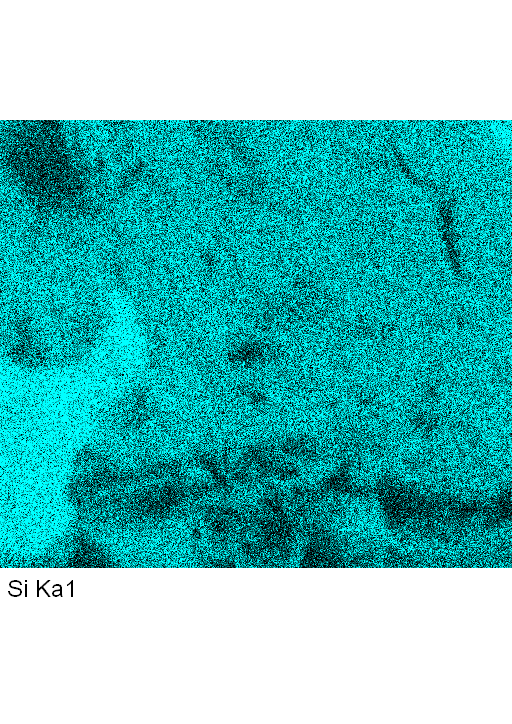

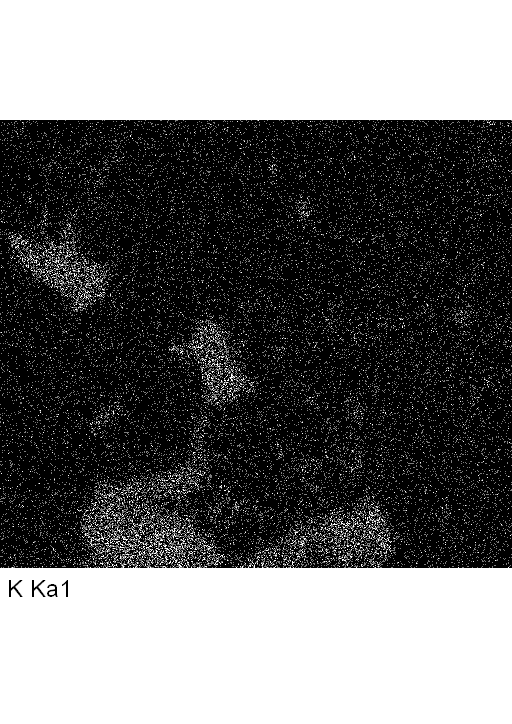

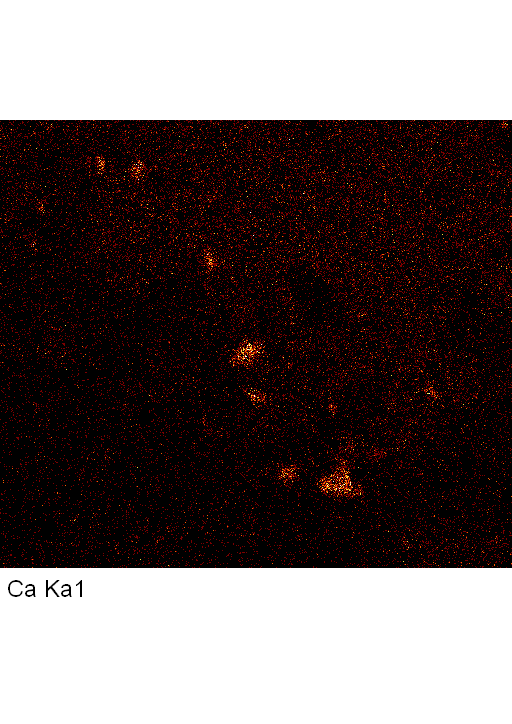


MAKMAL PENCIRIAN BAHAN BUMI (SEM/EDX/WDX)

20/02/2017 16:18:31

Sample: ODO-OWA

Type: Default

ID:

Spectrum processing :

Peaks possibly omitted : 4.471, 6.402 keV

Processing option : Oxygen by stoichiometry (Normalised)

Number of iterations = 2

Standard :

C CaCO3 1-Jun-1999 12:00 AM

Na Albite 1-Jun-1999 12:00 AM

Al Al2O3 1-Jun-1999 12:00 AM

Si SiO2 1-Jun-1999 12:00 AM

K MAD-10 Feldspar 1-Jun-1999 12:00 AM

Ca Wollastonite 1-Jun-1999 12:00 AM

| Element | Weight% | Atomic% | Compd% | Formula |  |
| --- | --- | --- | --- | --- | --- |
|  |  |  |  |  |  |
| C K | 1.04 | 1.76 | 3.82 | CO2 |  |
| Na K | 4.96 | 4.36 | 6.68 | Na2O |  |
| Al K | 9.68 | 7.25 | 18.28 | Al2O3 |  |
| Si K | 30.71 | 22.10 | 65.71 | SiO2 |  |
| K K | 2.16 | 1.12 | 2.61 | K2O |  |
| Ca K | 2.07 | 1.05 | 2.90 | CaO |  |
| O | 49.37 | 62.37 |  |  |  |
| Totals | 100.00 |  |  |  |  |
