# Supplementary material for: EDX-SEM-XRF data from selected Precambrian Basement Complex rock samples in part of Southwestern Nigeria
Source: Data Brief. 2018 Sep 8;20:1525–31. doi: 10.1016/j.dib.2018.09.014 (PMC6153388; doi:10.1016/j.dib.2018.09.014)
Supplement: Supplementary file 8 — Supplementary material [file mmc8.doc]

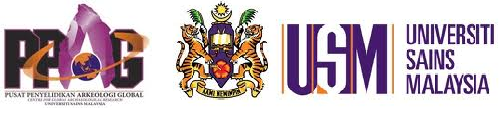

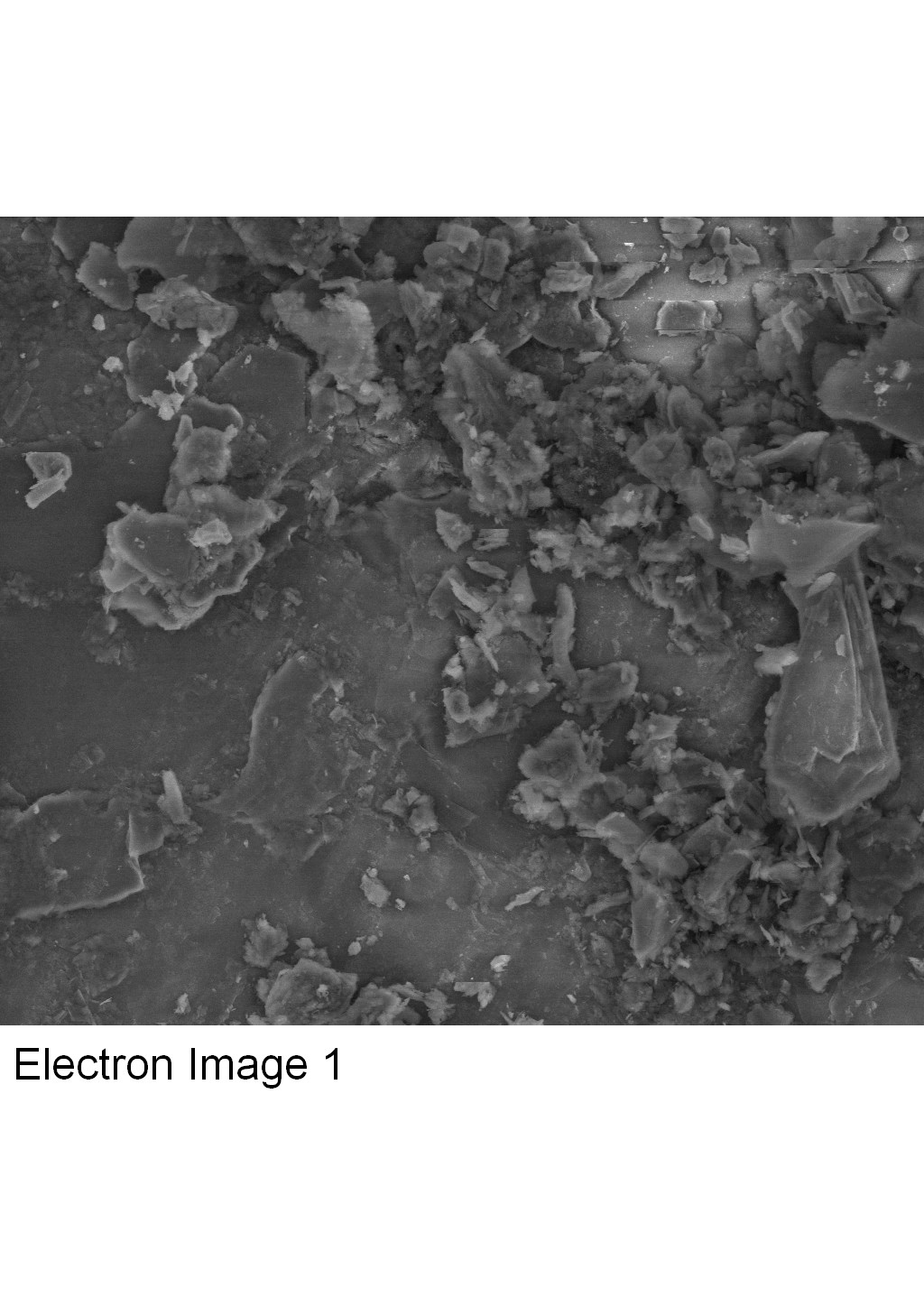

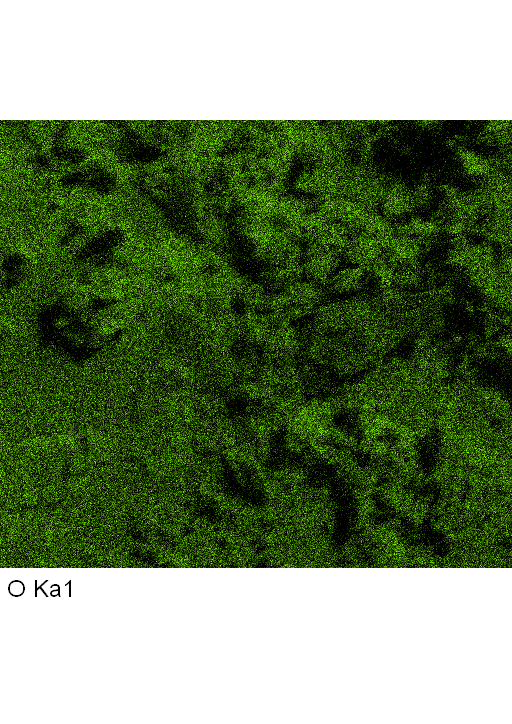

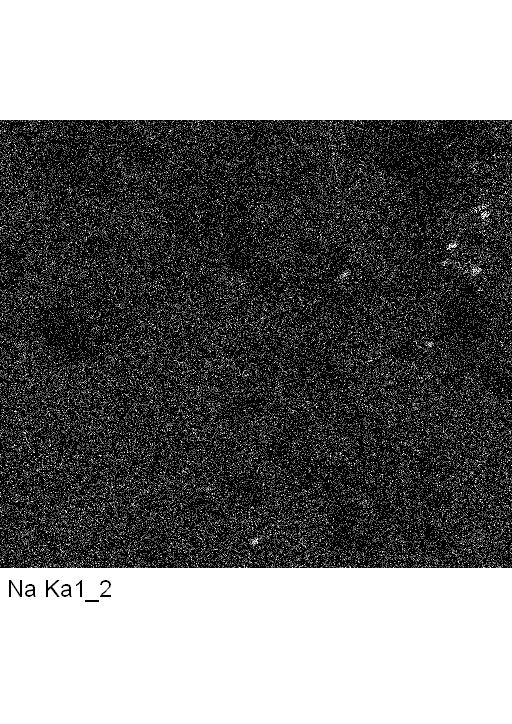

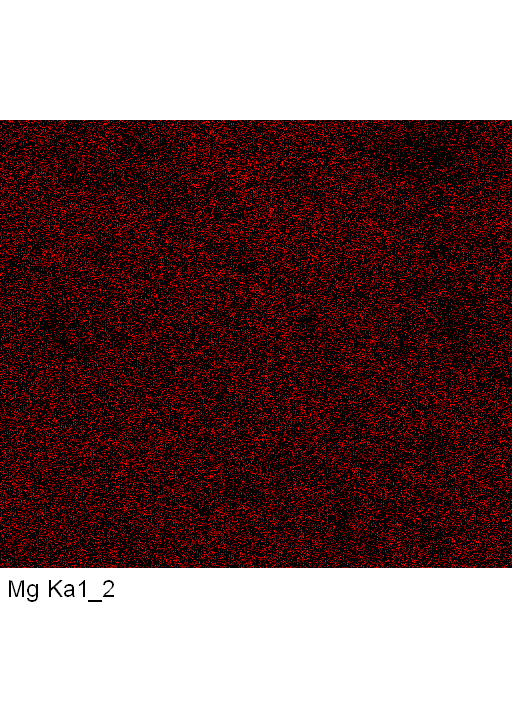

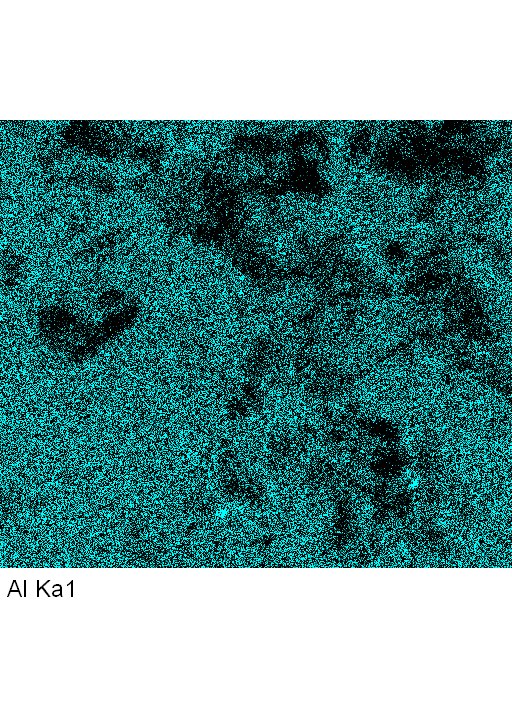

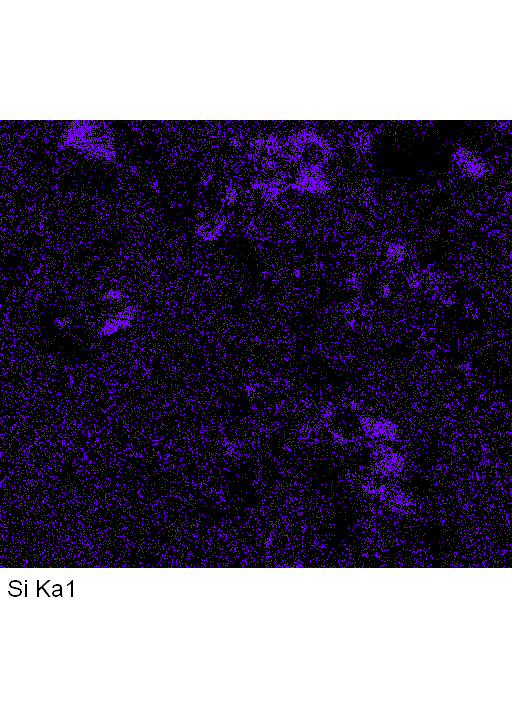

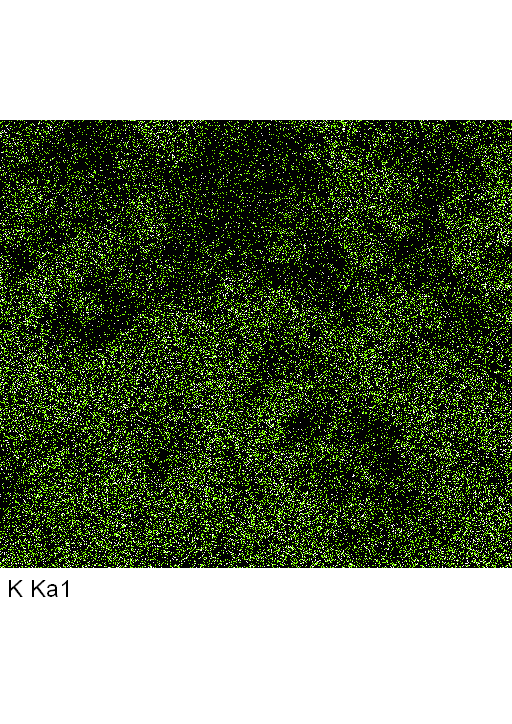

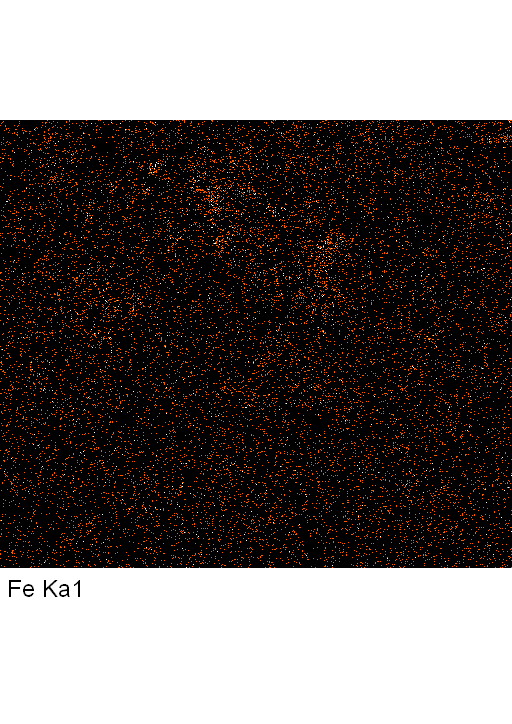


MAKMAL PENCIRIAN BAHAN BUMI (SEM/EDX/WDX)

24/03/2017 15:26:03

Sample: Sample 8

Type: Default

ID:

Spectrum processing :

Peak possibly omitted : 4.482 keV

Processing option : All elements analyzed (Normalised)

Number of iterations = 2

Standard :

O SiO2 1-Jun-1999 12:00 AM

Na Albite 1-Jun-1999 12:00 AM

Mg MgO 1-Jun-1999 12:00 AM

Al Al2O3 1-Jun-1999 12:00 AM

Si SiO2 1-Jun-1999 12:00 AM

K MAD-10 Feldspar 1-Jun-1999 12:00 AM

Fe Fe 1-Jun-1999 12:00 AM

| Element | Weight% | Atomic% |  |
| --- | --- | --- | --- |
|  |  |  |  |
| O K | 53.16 | 67.92 |  |
| Na K | 0.49 | 0.44 |  |
| Mg K | 0.20 | 0.17 |  |
| Al K | 15.80 | 11.97 |  |
| Si K | 21.00 | 15.28 |  |
| K K | 5.14 | 2.68 |  |
| Fe L | 4.21 | 1.54 |  |
|  |  |  |  |
| Totals | 100.00 |  |  |
