# Supplementary material for: EDX-SEM-XRF data from selected Precambrian Basement Complex rock samples in part of Southwestern Nigeria
Source: Data Brief. 2018 Sep 8;20:1525–31. doi: 10.1016/j.dib.2018.09.014 (PMC6153388; doi:10.1016/j.dib.2018.09.014)
Supplement: Supplementary file 9 — Supplementary material [file mmc9.doc]

Table 1. XRF data showing the weight percentage of the major oxide present in the selected rocks samples.

| **Sample** | **SiO_2_** | **TiO_2_** | **Al_2_O_3_** | **Fe_2_O_3_** | **MnO** | **MgO** |
| --- | --- | --- | --- | --- | --- | --- |
| **OMR-1** | 92.6376 | 0.1436 | 5.8576 | 1.0906 | 0.0059 | - |
| **OSI-1** | 72.6197 | 0.2656 | 14.6405 | 3.4597 | 0.048 | 0.4446 |
| **ORG-1** | 94.7572 | 0.0329 | 3.4189 | 1.4561 | 0.0087 | - |
| **RORE-1** | 92.3361 | 0.1078 | 5.6744 | 1.5596 | 0.0091 | 0.0101 |
| **IOD-1** | 50.3192 | 0.1284 | 34.6586 | 5.0739 | 0.0315 | 0.1248 |
| **IWS-1** | 96.1563 | 0.0624 | 1.5788 | 1.2672 | 0.0096 | 0.1335 |
| **OWS-1** | 70.8916 | 0.265 | 15.0143 | 3.9105 | 0.0425 | 0.2606 |
| **OWF-1** | 95.5966 | 0.1215 | 2.1644 | 1.2684 | 0.009 | 0.0415 |
| **ISS-1** | 93.3817 | 0.1259 | 4.548 | 1.5774 | 0.0115 | - |
| **ORK-1** | 5.0271 | 0.0701 | 0.5003 | 1.8887 | 0.0384 | 24.058 |
| **OKN-1** | 91.8008 | 0.087 | 5.2898 | 2.0026 | 0.0144 | - |
| **TLC-1** | 66.5152 | 0.05 | 1.9786 | 7.3537 | 0.0189 | 22.2975 |

Table 1. Continue

| **Sample** | **CaO** | **Na_2_O** | **K_2_O** | **P_2_O_5_** |
| --- | --- | --- | --- | --- |
| **OMR-1** | 0.0425 | - | 0.0382 | 0.0206 |
| **OSI-1** | 1.2427 | 0.7598 | 6.0245 | 0.0672 |
| **ORG-1** | 0.0554 | 0.0185 | 0.0932 | - |
| **RORE-1** | 0.0511 | - | 0.0306 | 0.0167 |
| **IOD-1** | 0.0355 | 0.2668 | 8.6569 | 0.0539 |
| **IWS-1** | 0.0713 | 0.0123 | 0.3693 | 0.0167 |
| **OWS-1** | 1.9705 | 0.7169 | 6.2578 | 0.1817 |
| **OWF-1** | 0.2001 | 0.0176 | 0.0577 | 0.0132 |
| **ISS-1** | 0.0531 | - | 0.0782 | 0.0109 |
| **ORK-1** | 67.7985 | - | 0.0796 | 0.0737 |
| **OKN-1** | 0.091 | 0.0119 | 0.3635 | 0.0485 |
| **TLC-1** | 0.0402 | - | 0.0083 | - |

Table 1. Continue

| **Sample** | **SO_3_** | **Cr_2_O_3_** | **NiO** | **CuO** | **ZnO** | **Rb_2_O** | **ZrO2** | **PbO** |
| --- | --- | --- | --- | --- | --- | --- | --- | --- |
| **OMR-1** | 0.0166 | 0.0783 | 0.0059 | 0.003 | 0.005 |  | 0.0393 | 0.0097 |
| **OSI-1** | 0.0231 | 0.0733 | 0.0068 | 0.0048 | 0.011 | 0.0629 | 0.0478 | 0.0183 |
| **ORG-1** | 0.0147 | 0.1089 | 0.0068 | 0.0042 | 0.0051 |  | 0.0107 | 0.0086 |
| **RORE-1** | 0.025 | 0.1161 | 0.0075 | 0.0043 | 0.007 |  | 0.0275 | 0.0138 |
| **IOD-1** | 0.013 | 0.0308 |  |  | 0.029 | 0.3443 | 0.0035 | 0.0068 |
| **IWS-1** | 0.0879 | 0.1091 | 0.0076 | 0.0043 | 0.0302 | 0.003 | 0.0235 | 0.057 |
| **OWS-1** | 0.0141 | 0.0727 | 0.0075 | 0.0045 | 0.0098 | 0.0314 | 0.0362 | 0.0118 |
| **OWF-1** | 0.1884 | 0.0977 | 0.0075 | 0.0063 | 0.0625 | 0.0012 | 0.0283 | 0.1165 |
| **ISS-1** | 0.0401 | 0.1053 | 0.0066 | 0.0038 | 0.0115 |  | 0.0195 | 0.024 |
| **ORK-1** | 0.1219 |  | 0.0146 |  | 0.0907 | 0.0046 |  | 0.167 |
| **OKN-1** | 0.0575 | 0.1347 | 0.0075 | 0.0051 | 0.0176 | 0.0023 | 0.0232 | 0.0354 |
| **TLC-1** | 0.0068 | 1.3161 | 0.3729 |  | 0.021 |  |  | 0.0052 |

Table 2. XRF data showing the weight percentage of oxide of the trace elements present in the selected rocks samples.

| **Sample** | **SrO** | **Y_2_O_3_** | **Ga_2_O_3_** | **BaO** | **Cl** | **Nb_2_O_5_** | **Ag_2_O** | **Ta_2_O_5_** | **Co_2_O_3_** |
| --- | --- | --- | --- | --- | --- | --- | --- | --- | --- |
| **OMR-1** | 0.0022 | 0.0032 | - | - | - | - | - | - | - |
| **OSI-1** | 0.0327 | 0.0239 | 0.004 | 0.112 | - | - | - | - | - |
| **ORG-1** | - | - | - | - | - | - | - | - | - |
| **RORE-1** | - | 0.0033 | - | - | - | - | - | - | - |
| **IOD-1** | 0.0019 | - | 0.0214 | - | - | 0.0899 | 0.0983 | 0.0116 | - |
| **IWS-1** | - | - | - | - | - | - | - | - | - |
| **OWS-1** | 0.1108 | 0.0086 | 0.0032 | 0.1644 | 0.0084 | - | - | - | - |
| **OWF-1** | 0.0014 | - | - | - | - | - | - | - | - |
| **ISS-1** | - | 0.0025 | - | - | - | - | - | - | - |
| **ORK-1** | 0.6668 | - | - | - | - | - | - | - | - |
| **OKN-1** | 0.0074 | - | - | - | - | - | - | - | - |
| **TLC-1** | - | - | - | - | - | - | - | - | 0.0153 |

Table 3. XRF data of the major oxide present in the selected rocks samples.

| **Sample** | **Al_2_O_3_/SiO_2_** | **SiO_2_/Al_2_O_3_** | **Al_2_O_3_/TiO_2_** | **ICV** |
| --- | --- | --- | --- | --- |
| **OMR-1** | 0.0632 | 15.849 | 40.7912 | 0.201 |
| **OSI-1** | 0.2016 | 4.9602 | 55.1224 | 0.8182 |
| **ORG-1** | 0.0361 | 27.7157 | 103.9179 | 0.4773 |
| **RORE-1** | 0.0615 | 16.2724 | 52.6382 | 0.2927 |
| **IOD-1** | 0.6888 | 1.4519 | 269.9268 | 0.4094 |
| **IWS-1** | 0.0164 | 60.9047 | 25.3013 | 1.1801 |
| **OWS-1** | 0.2118 | 4.7216 | 56.6577 | 0.8764 |
| **OWF-1** | 0.0226 | 44.1677 | 17.814 | 0.7366 |
| **ISS-1** | 0.0487 | 20.5325 | 36.1239 | 0.3782 |
| **ORK-1** | 0.0995 | 10.0482 | 7.137 | 187.6138 |
| **OKN-1** | 0.0576 | 17.3543 | 60.8023 | 0.4695 |
| **TLC-1** | 0.0298 | 33.6173 | 39.572 | 15.02 |

Table 4. Data for the weight percentage of the oxide of the radioactive element present in the selected rocks samples.

| **Sample** | ThO_2_ |
| --- | --- |
| OMR-1 |  |
| OSI-1 | 0.0073 |
| ORG-1 |  |
| RORE-1 |  |
| IOD-1 |  |
| IWS-1 |  |
| OWS-1 | 0.0051 |
| OWF-1 |  |
| ISS-1 |  |
| ORK-1 |  |
| OKN-1 |  |
| TLC-1 |  |
